# Assembly of synthetic Aβ miniamyloids on polyol templates

**DOI:** 10.3762/bjoc.11.284

**Published:** 2015-12-17

**Authors:** Sebastian Nils Fischer, Armin Geyer

**Affiliations:** 1 Faculty of Chemistry, Philipps University Marburg, Hans-Meerwein-Straße 4, 35032 Marburg, Germany

**Keywords:** Alzheimer, boronic acid, miniamyloids, NMR spectroscopy, peptides

## Abstract

Covalent dynamic chemistry is used to mimic the first steps of the highly cooperative fibril formation of Aβ peptides. For that purpose, Aβ peptide pentapeptide boronic acids **1** and **2** were synthesized by solid-phase peptide synthesis and studied in esterification experiments with polyhydroxylated templates. The bis-hydroxylated dipeptide Hot=Tap serves as a template of adjustable degree of oligomerization which spontaneously forms boronic esters with peptides of type **1** and **2**. Nuclear magnetic resonance can differentiate between regioisomeric boronic esters and identifies preferred sites of esterification on the dimeric template **9**. 2-Formylphenylboronic acid (**14**) is used to link the parent pentapeptide Leu-Val-Phe-Phe-Ala to the template **16** to obtain threefold boronic ester **17**. The miniamyloid **17** assembles from seven components by imine and boronic ester bonds between the peptides and the template. The relative orientation and spacing of the peptides mimic the assembly of peptides in Alzheimer β-amyloids.

## Introduction

**Figure 1:**
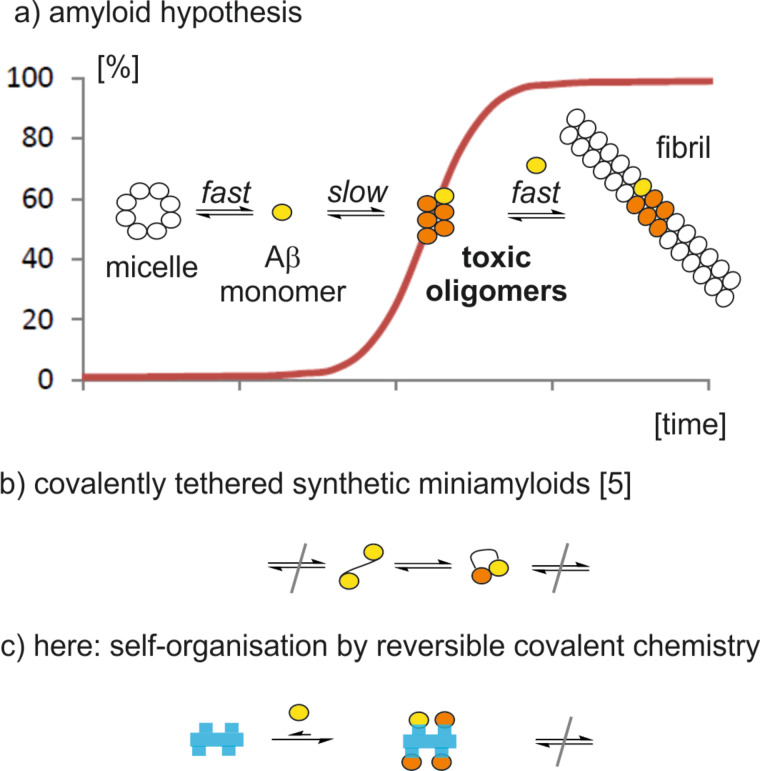
a) Schematic representation of the Aβ fibril formation. The monomeric peptide is shown as a colored ball to indicate the unfolded (yellow) or the β-sheet (orange) conformation. Micellar aggregates of Aβ are in equilibrium with dissolved monomers. The autocatalytic and irreversible fibril formation starts after an induction phase with a sigmoidal behavior. b) Covalent tethering of Aβ peptides mimics the first step of fibril formation. Additional charged amino acids prevent the further aggregation of covalently tethered oligomers. c) The reversible covalent tethering of Aβ peptides on an organic template (blue bar) is topic of the present study. The spacing of attachment sites supports the cooperative interaction between Aβ peptides, while the size of the template limits the aggregation degree.

Aβ peptides spontaneously form amyloid fibrils which are a major component of Alzheimer plaques [1]. The cooperative thermodynamically-driven process of fibril formation has an induction period which depends on the conditions of amyloid formation [2]. Alternatively, it is started by seeding with amyloid fragments whose large surface-to-core ratio greatly accelerates the fibril formation. Although amyloid structures are well studied in vitro, their in vivo relevance at the onset of Alzheimer’s disease remains under debate [3]. Aβ peptides are known to form fibrils and there is no way to stop the process until complete precipitation. Intermediate soluble oligomers were identified as neurotoxic agents, but they are difficult to study because of their heterogeneous composition and transient character due to the onset of amyloid formation (Figure 1) [4]. Based on the concept of the existence of toxic Aβ oligomers, we recently developed covalently linked dimers of Aβ epitopes – so called synthetic Aβ miniamyloids – and successfully characterized their neurotoxicity [5]. These oligomeric peptides do not show an unmitigated fibrillation. The dimeric Aβ(28–40) epitopes exhibit reversible folding and show avidity towards the conformation-specific nAbsAβ, the antibody which selectively binds and eliminates toxic Aβ oligomers but neither binds Aβ monomers or fibrils. The Aβ(28–40) epitopes were irreversibly linked at their carboxy-terminal ends to the two amine groups of a lysine. Only covalently linked dimeric peptides with parallel peptide strands showed a cooperative folding behavior. The correct relative orientation of the individual peptide strands proved to be crucial for their cooperative and reversible un/folding behavior to mimic the first step of Aβ oligomerization. In the present article, we further develop the idea of systematic variation of the oligomerization degree of Aβ fragments by using dynamic covalent chemistry for the assembly of miniamyloids. The size of a polyol template will limit the aggregation degree of Aβ peptides, which are linked to the template as boronic esters. A shape-persistent template is necessary which offers the correct spacing between Aβ strands to allow for their preferred cross-β-sheet contacts. The modular assembly of peptides on a template is expected to access synthetic Aβ miniamyloids with molecular weights in the region of several kilodalton.

**Figure 2:**
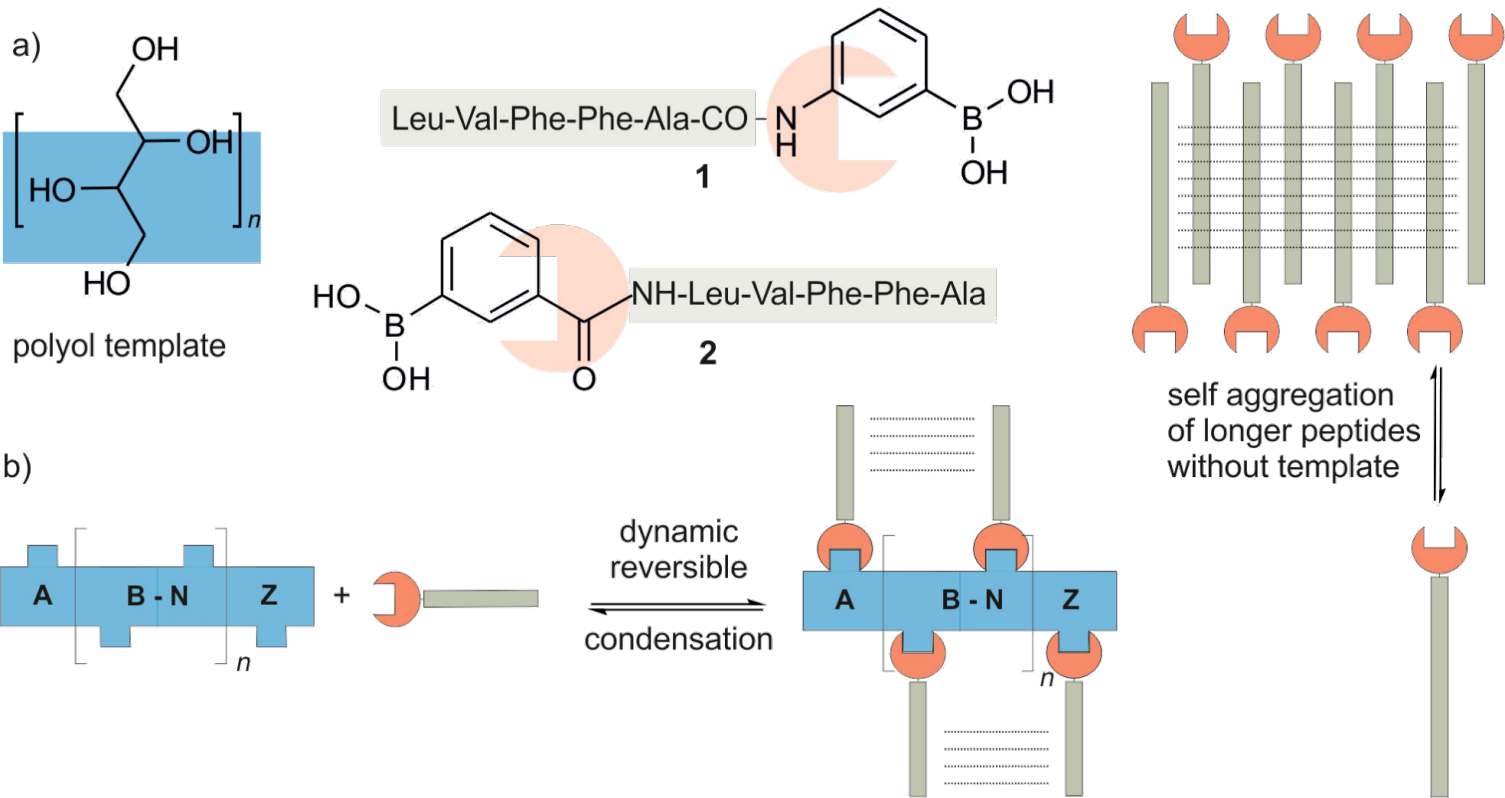
a) Peptide boronic acids **1** and **2** are schematically shown as green sticks (peptide) with a red gripper (boronic acid) and the template is blue. The peptide must be short enough to prevent the fibrillation shown on the right. b) Non-covalent interactions between neighboring peptides on the template support esterification. The fine tuning of Aβ aggregation is performed by tailoring the peptide length to match the template size.

Sugars (polyols) were already investigated as templates for peptides [6] and vice versa [7] but both concepts were not yet used for the assembly of monodisperse Aβ miniamyloids. Lehn’s concept of constitutional dynamic chemistry (CDC) [8–9] relies on chemical bonds which equilibrate under the chosen reaction conditions to form the thermodynamically most stable product. Constitutional dynamic chemistry has emerged as a versatile tool for the synthesis of complex molecular structures. It takes advantage of the reversible nature of bond formation, for example, disulfide [10], acetal [11], imine [12] and boronates [13–16], to allow the generation of new covalent structures under thermodynamic control. Complete esterification is observed for boronate esters bearing *ortho*-amines [17]. The conceptual advantage compared to traditional irreversible chemistry is the self-correcting reversibility if individual peptides which are bound in the wrong orientation resulting in a strong preference for defined ring sizes [18] or oligomerization degrees [19]. The idea appears especially attractive for the assembly of peptides with high aggregation tendencies, such as those forming amyloids. The solubility of the assembly should be maintained throughout the experiment and precipitation strictly avoided to reach the thermodynamic equilibrium with the formation of the most stable miniamyloid in solution. Not all functional groups listed above for the reversible dynamic chemistry of specific oligomers are suitable for oligopeptides. Disulfide bonds seem to be the natural choice for the reversible covalent chemistry of peptides, but it appeared to us impossible to prevent both the template and the peptide from homo-oligomer formation. Self-aggregation is avoided only when two different reactive groups are employed; that is why the reversible esterification between boronic acids and diols appeared an attractive solution to us. In a first approach, we planned to condense short peptide boronic acids with polyol templates for the assembly of an Aβ-dimer. The concept of tailoring the length of the peptide boronic acid and a polyol template is shown in Figure 2.

## Results and Discussion

The shortest known functional expansion of the amyloidogenic Aβ-peptide is the β-amyloid (17–21) Leu-Val-Phe-Phe-Ala [20] which was investigated as both a C-terminal **1** and as an N-terminal boronic acid **2**. Peptide boronic acids of type **1** were synthesized on polymer-bound diethanolamine (PS-DEAM resin), according to the protocol in Supporting Information File 1, Figure S1 [21]. The electron-poor boronic acid **2**, which was expected to be more reactive in boronic ester formation, was obtained by routine solid-phase peptide synthesis on chloro-(2'-chloro)trityl polystyrene (CTC resin) and coupling of the unprotected boronic acid as the final building block. The C-terminal boronic acids need careful exclusion of water because of the reversible linkage to the resin, while no special precautions were necessary for peptides of type **2**.

**Figure 3:**
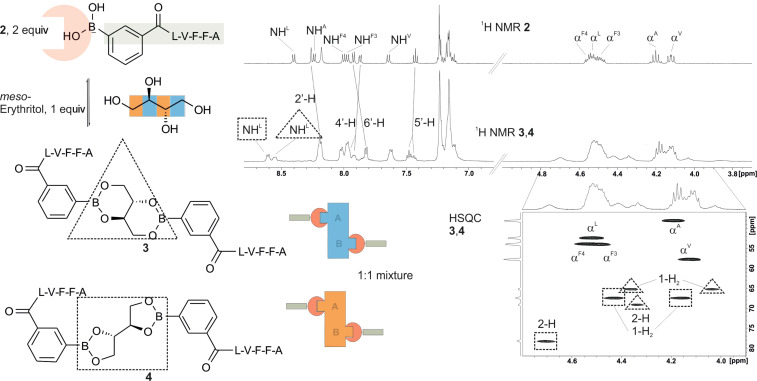
*Meso*-erythritol is a promiscuous polyol which forms mixtures of esters due to the formation of 5-membered (square, **4**) and 6-membered (triangle, **3**) esters. The overlapping resonances in the ^1^H NMR spectra of the esters in the region between 4 and 5 ppm are resolved in the HSQC spectrum, where the alpha-carbons are visible below 50 ppm and the CH–OB groups between 65 and 80 ppm.

The template should match the spacing of about 4 Å between the Aβ peptides within the Aβ fibrils [22]. Sugars appear as structurally diverse polyfunctional templates, but the problem of regioselective esterification is shown in Figure 3. The condensation of *meso*-erythritol with a peptide boronic acid is complete in dimethyl sulfoxide, but a nearly equimolar mixture of the regioisomeric 5- and 6-membered ester is obtained. The CH and CH_2_ protons of the polyol and the CH^α^ overlap between 4 and 5 ppm in the ^1^H NMR, however, they are well separated in a CH correlation. Nuclear magnetic resonance spectroscopy is the analytical method of choice not only to characterize the conversion of template and peptide or to differentiate regioisomers, but also because it can detect noncovalent interactions between peptide strands by NOE contacts or other techniques.

**Figure 4:**
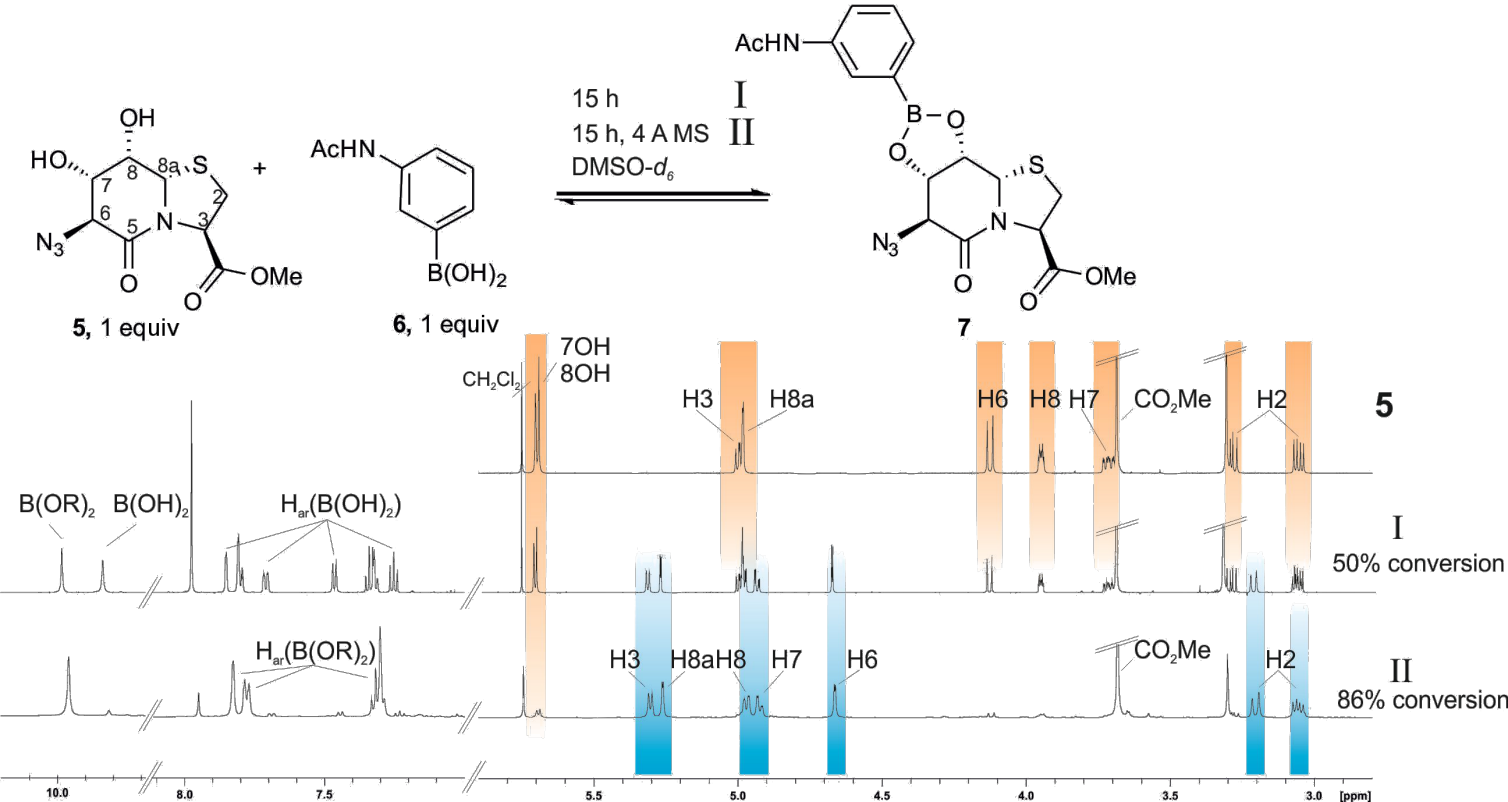
Diol **5** (6.08 mg, 20.0 μmol, 1.0 equiv) and 3-(acetylamino)phenylboronic acid (**6**, 3.56 mg, 20.0 μmol, 1.0 equiv) form ester **7** in an NMR experiment in DMSO-*d*_6_. The ^1^H NMR spectrum shows an esterification of 58% (2.5 equiv H_2_O). Molecular sieves which adsorb water out of the equilibrium push the equilibrium to 86% (1.0 equiv H_2_O) conversion.

The diols on the template must be separated from each other far enough to exclude the simultaneous formation of mixtures of 5- and 6-membered boronic esters. However, the oligosaccharides, which are available with a consistent oligomerization degree of dimer, trimer, tetramer and higher, are known to have only low tendencies of boronic ester formation [23]. Therefore, we turned to *cis*-dihydroxylated **5**, which exhibited a unique reactivity towards boronic acids because it forms two anellated *cis*-fused rings. **5** shows 86% esterification after drying in a 1:1 mixture with the arylboronic acid **6**, a single ester even under the dilution conditions of an NMR tube (Figure 4). The signal set of boronic ester **7** is significantly shifted compared to the ^1^H NMR spectra of the educts, because the central δ-valerolactam of the 5,6,5-membered tricyclic fused ring system is locked in the boat conformation. Increasing the measuring temperature promotes higher conversion rates (Supporting Information File 1, Figure S2). Contrarily, the addition of water promotes the hydrolysis of the boronic ester. The high but incomplete esterification is a good basis for the analysis of noncovalent interactions between peptide strands, which are expected to push the esterification towards quantitative conversion.

**Figure 5:**
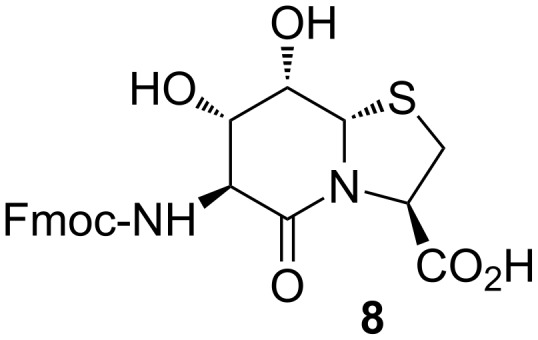
Fmoc-Hot=Tap-OH (**8**).

Compound **5** combines side-chain *cis*-diol functionality for boronic ester formation and the potential of controlled oligomerization by peptide chemistry. Azido ester **5** is a precursor of the dipeptide Hot=Tap, which serves as a β-turn mimic in peptides and proteins [24–26]. Alternatively, Fmoc-Hot=Tap-OH (**8**, Figure 5) is available for oligomerization on SPPS to templates of adjustable length with a systematic increase of the number of *cis*-diol functions assembled on a rigid peptide backbone. In solution, instead of a protecting group, the azide serves as a precursor of the amine which is necessary for the fragment coupling of **5** to oligomeric templates. Along this strategy, tetrapeptide **9** was obtained which represents a bis-dihydroxylated template. Hot=Tap oligomers are suitable for NOE-sequencing along the protons NH(Hot*^i^*)-H8a(Hot*^i^*)-H3(Tap*^i^*^+1^)-NH(Hot*^i^*^+2^) trace in the same way as it is normally performed for NH*^i^*-Hα*^i^*-NH*^i^*^+1^ of peptides.

**Figure 6:**
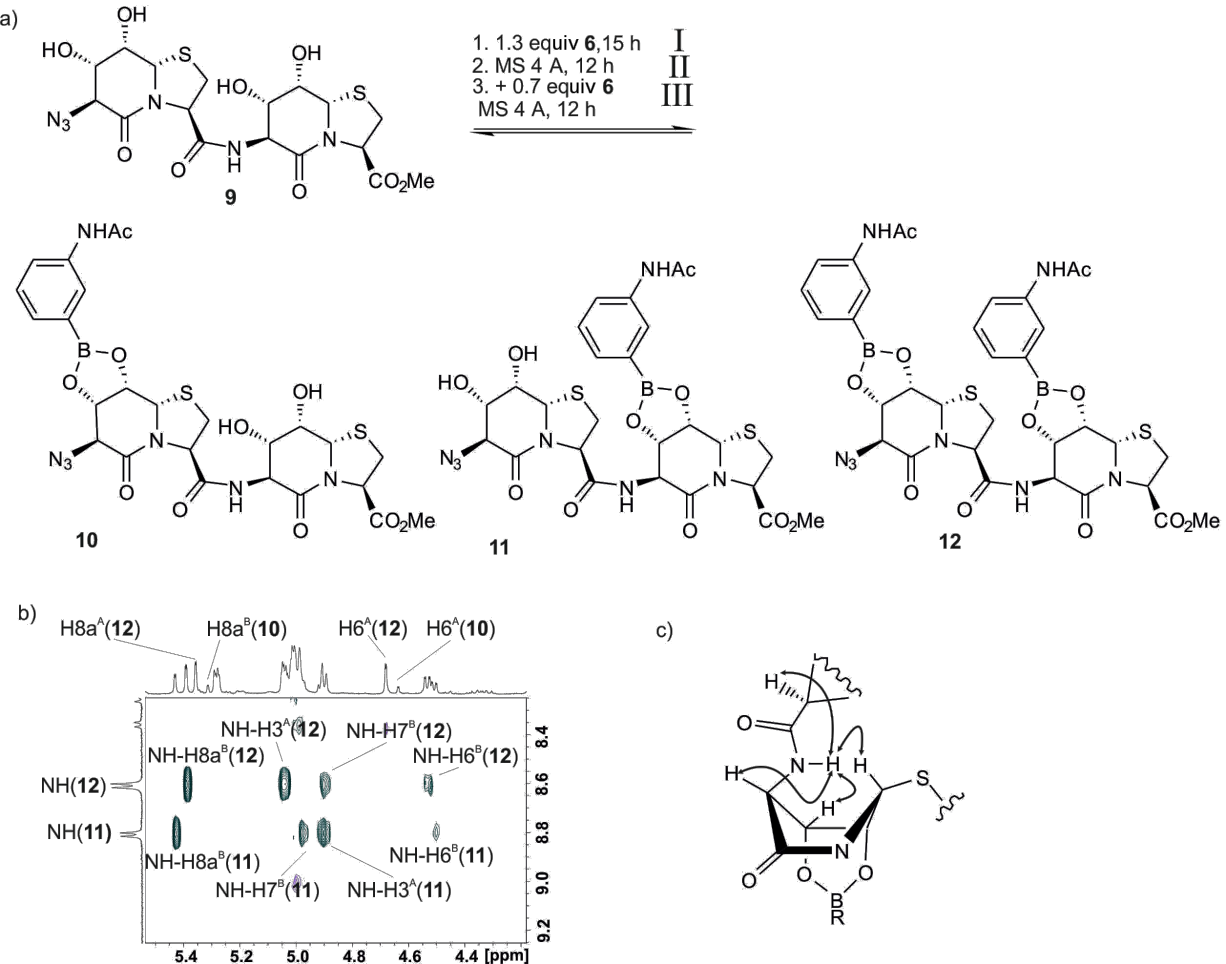
Template **9** and boronic acid **6** can form the monoesters **10** and **11**, or the diester **12**. The signal assignments were performed by homo- (DQF-COSY, TOCSY) and heteronuclear (HSQC, HMBC) 2D NMR methods. The NOESY spectrum identifies the position and the relative orientation of the aryl group of the boronic ester to the valerolactam ring of Hot.

Together with the chemical shift information about the esterification sites (Figure 2), a differentiation between the two possible monoesters of the Hot=Tap dimer **10** and **11** is possible (Figure 6). Addition of 0.7 equivalents of boronic acid to the dimeric template **9** intentionally leads to incomplete esterification and identifies the C-terminal site B as the slightly preferred esterification site. This technique is relevant for identifying hydrophobic clustering of peptides on longer Hot=Tap oligomers. Table 1 shows the progress of esterification between template **9** and aniline boronic acid **6**. The ratio between monoesters **10** to **11** remains steadily 1:2 until approximately 1.2 equivalents of boronic acid, while the amount of diester increases constantly.

**Table 1:** Signal integration of ^1^H NMR spectra give a quantitative measure of the relative amounts of esters under the conditions specified in Figure 6. Experimental details are given in Supporting Information File 1.

	**9**	**10**	**11**	**12**	equiv **6**

**I**	0.38	0.14	0.33	0.15	0.7
**II**	0.17	0.13	0.30	0.40	1.2
**III**	0	0	0.18	0.82	1.8

**Figure 7:**
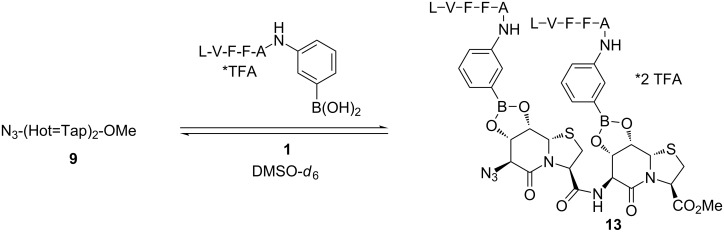
Template **9** (2.30 mg, 4.40 µmol, 1.0 equiv) and peptide boronic acid **1** (7.35 mg, 8.80 µmol, 2.0 equiv) were dissolved in 0.7 mL DMSO-*d*_6_ in a NMR tube. A ratio of **9**/C-terminal monoester/N-terminal monoester/**13** (0.04:0.24:0.08:0.60) was observed in the presence of 5.5 equiv of water.

A quantitative esterification of boronic acids needs the exclusion of water, which becomes more difficult for larger hydrophilic molecules such as polyols ond oligopeptides. In order to investigate this question, (Hot=Tap)_2_ was also esterified with peptide boronic acid **1**. No cooperativity is expected because the Hot=Tap dimer orients the two peptides in opposite directions due to the β-turn character of the individual Hot=Tap template (Figure 7).

**Figure 8:**
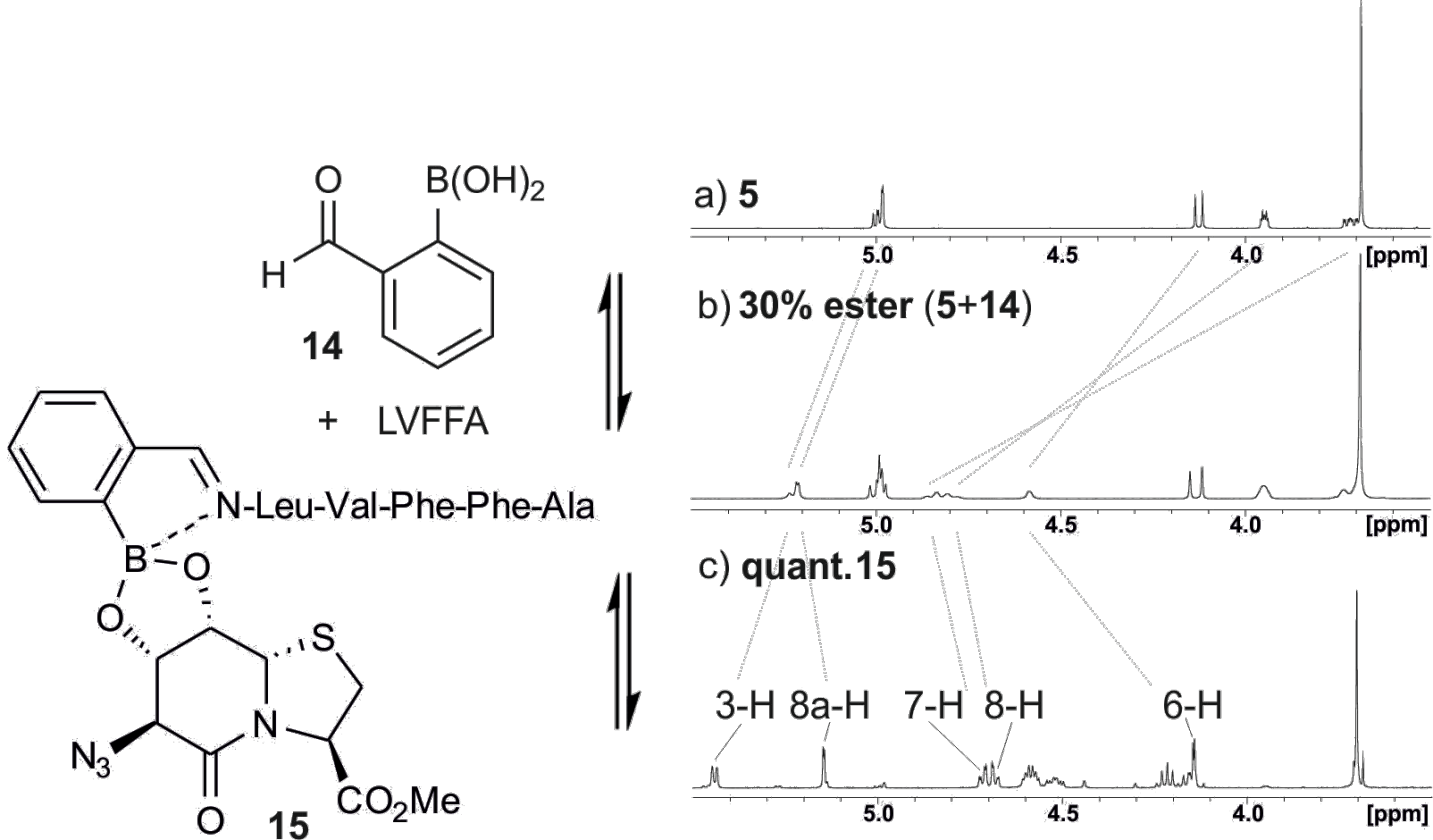
Template **5** (^1^H NMR expansion shown for reference DMSO-*d*_6_, 300 K) and peptide Leu-Val-Phe-Phe-Ala are linked by 2-formylphenylboronic acid (**14**) in an NMR experiment (DMSO-*d*_6_, 300 K). The esterification of **5** with boronic acid is incomplete and stops at 30% conversion. The addition of the peptide which forms the imine drives the esterification to completion (**15**).

The synthesis of peptide boronic acids appeared cumbersome; that is why we investigated the assembly of a three-component system of unmodified peptide, 2-formylphenylboronic acid and the Hot=Tap oligomer. The mixture of azide **9**, 2-formylphenylboronic acid (**14**) and LVFFA shows a single signal set for the esterified product (Figure 8). Another advantage of this system is the cooperativity of imine formation and esterification, because the imino nitrogen coordinates the boron atom and shifts the esterification equilibrium towards higher conversion rates.

**Figure 9:**
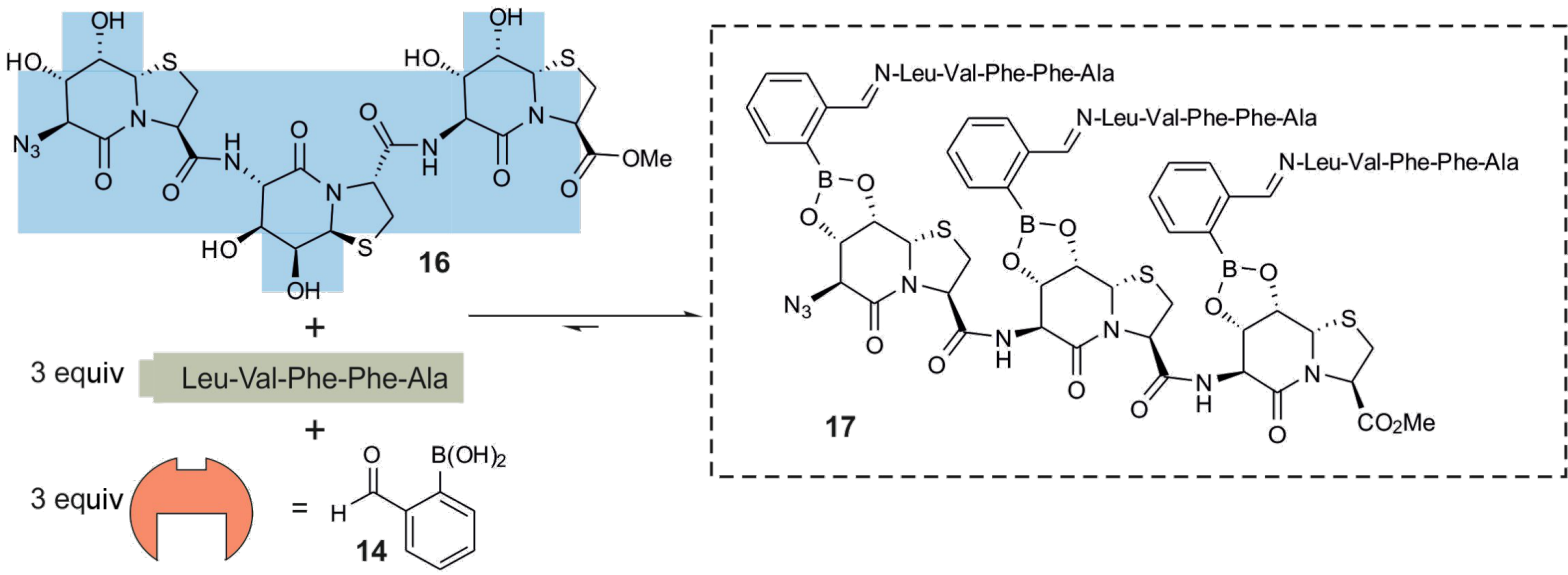
The trimeric template **16** together with 3 equivalents of pentapeptide LVFFA and 2-formylphenylboronic acid (**14**) forms trimer **17**. The equilibrium lies far on the side of the triple ester in spite of the 9 equivalents of water which are released during the conversion in DMSO-*d*_6_ at 300 K.

Encouraged by the observation of the monomeric Hot=Tap, we performed the same for dimeric and trimeric Hot=Tap **16** (Figure 9). Only the final spectrum is shown as Supporting Information File 1, Figure S4 because of the unmanageable number of several dozen possible titration intermediates. Miniamyloid **17** already has a molecular mass of nearly 3 kDa. The β-turn-type backbone orients neighboring peptides in opposite directions, giving every third peptide a parallel orientation with a spacing amenable for cross β-sheet formation. In spite of the two condensation steps per peptide (imine formation and boronic ester formation), a high conversion rate is observed in the ^1^H NMR.

The triple ester remained stable in the NMR tube in spite of the slow addition of water to the imine and formation of hemiaminals, which appeared as minor signal sets in the proton ^1^H NMR spectra. Detailed analytical studies of **17** and its possible functional mimicry of toxic Aβ amyloids, according to our previous study of irreversibly covalently linked miniamyloids, are under progress.

## Conclusion

In conclusion, we presented a strategy which merges the supramolecular chemistry of Aβ with the concepts of reversible covalent chemistry. The present study shows how oligomers of the Hot=Tap dipeptide serve as templates for the reversible covalent esterification of boronic acids which mediate the assembly of monodisperse Aβ miniamyloids. The modular synthesis of the trimer **17** is significantly more efficient than the traditional covalent irreversible assembly of Aβ miniamyloids described in [5].

## Supporting Information

File 1 Experimental part.
