# Supplementary material for: Assembly of synthetic Aβ miniamyloids on polyol templates
Source: Beilstein J Org Chem. 2015 Dec 17;11:2646–53. doi: 10.3762/bjoc.11.284 (PMC4685884; doi:10.3762/bjoc.11.284)
Supplement: File 1 — Experimental part. [file Beilstein_J_Org_Chem-11-2646-s001.pdf]

Supporting Information

for

Assembly of synthetic Aβ miniamyloids on polyol templates

Sebastian Nils Fischer and Armin Geyer*

Address: Faculty of Chemistry, Philipps University Marburg, Hans-Meerwein-Straße 4, 35032 Marburg, Germany

Email: Armin Geyer - geyer@staff.uni-marburg.de

*Corresponding author

Experimental part

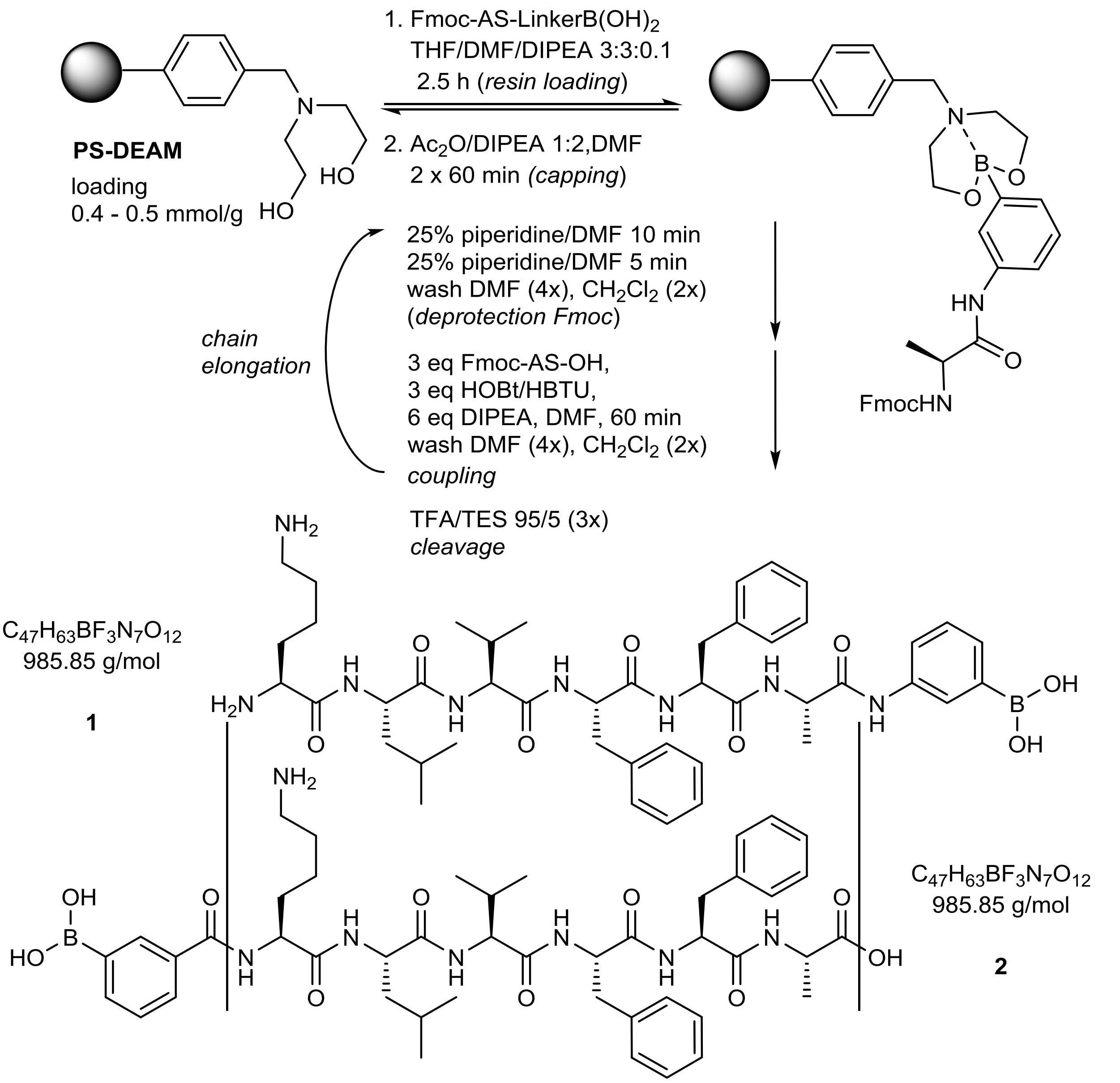

Figure S1: Reaction conditions for peptide synthesis on diethanolamine-functionalized polystyrene (PS-DEAM resin) for the assembly of peptides with C-terminal boronic acid. Inert reaction conditions are necessary because the labile linkage between boronic acid and resin. Fmoc deprotection with piperidine (25%) in DMF and final cleavage from the resin with TFA were performed according to standard SPPS.

General

All reagents were obtained from commercial sources and were used as received. Solvents were dried and distilled prior to use. NMR analyses were carried out on Bruker Avance 300–600 MHz spectrometers in deuterated DMSO or water as solvent. Peptides were freshly solubilized in DMSO-d_6 or 10 mM potassium hydrogen phosphate buffer pH 7/D_2O (5:1) at 30 °C under ultra-sonication and were directly investigated. The aqueous samples were centrifuged (10.000 rpm, 5 min) prior to use. Water suppression was achieved by excitation sculpting with gradients (double Watergate DPFGSE sequence). Signal assignment was achieved using 2D homo- (COSY, TOCSY, NOESY) and heteronuclear (HSQC, HMBC) experiments. The chemical shifts (δ) for carbon and proton are given compared to the residual solvent peak and are expressed in ppm.

Loading of the PS-DEAM resin for the synthesis of compound 1

N-Fmoc-alanyl-phenylboronic acid (**S1**) was loaded on the PS-DEAM resin under exclusion of oxygen and moisture according to the following flow diagram: swelling of the resin in DMF_{abs} (10 min); loading with 1.5 equiv **S1** in DMF/THF/DIPEA (3:3:0.1) (2.5 h); washing with DMF (3×); capping with DIPEA/Ac_2O (2:1) (60 min) washing with DMF (4×) and THF (2×) and dry under high vacuum.

Loading of 2-Cl-2-trityl resin for the synthesis of compound 2

Flow diagram: swelling of the resin in DMF_{abs} (10 min); loading with 1.2 equiv **Fmoc-Ala-OH** and 5 equiv DIPEA in DMF (2 h); washing with DMF (3×) and DCM (2×), capping with DCM/MeOH/DIPEA (80:15:5) (60 min) washing with DCM (2×) and dry under high vacuum.

Solid-phase peptide synthesis

The batch size was 0.1 mmol. Flow diagram: swelling of the resin in DMF_{abs} (10 min); deprotection 20% piperidine in DMF (10 min 1×, 5 min 1×); washing with DMF (4×) and DCM (2×), coupling with 3 equiv Fmoc Xaa-OH, HOBt, HBTU, and 6 equiv DIPEA in DMF (60 min); washing with DMF (4×) and DCM (2×).

Cleavage from the resin

The CTC-resin was treated with TFA/H_2O 95:5 for 1 h and the washed twice with the solvent. The PS-DEAM resin was treated with a mixture of DMF/water. The solution was concentrated under high vacuum. Then the peptide was precipitated with cold Et_2O_{abs}. The peptides were lyophilized from acetonitrile/water and purified if necessary by semipreparative HPLC.

(2'S)-meta-{N-[2-(N'-(9-Fluoroenylmethyloxycarbonyl))amino]propylcarbonyl}aminophenylboronic acid (S1)

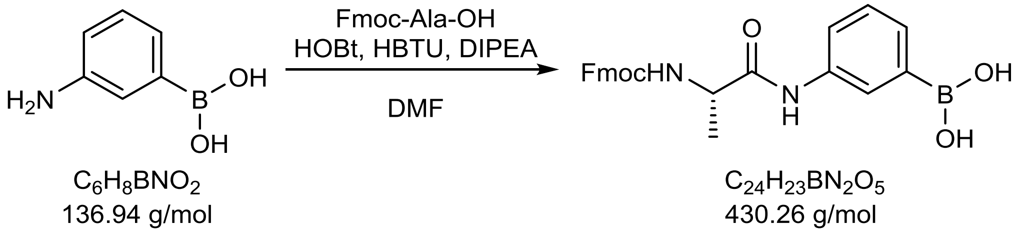

S1

Fmoc-Ala-OH (736 mg, 2.37 mmol, 1.1 equiv) was dissolved in DMF (21 mL) at 0 °C. HOBt (407 mg, 3.02 mmol, 1.4 equiv), HBTU (1.14 g, 3.01 mmol, 1.4 equiv) and DIPEA (1.28 mL, 7.54 mmol, 3.5 equiv) were added under stirring. *meta*-aminophenylboronic acid hemisulfate (400 mg, 2.15 mmol, 1.0 equiv) was added after 10 min and the residue was stirred for 16 h. The solution was diluted with EtOAc and washed with 1 N HCl and brine. The combined organic phases were dried ($MgSO_4$) and the solvent was removed. Flash chromatography (DCM/MeOH 20:1 $\rightarrow$ 9:1) yielded boronic acid **S1** (640 mg, 1.49 mmol, 69%) as a colourless powder. **Tlc:** R_f = 0.53 (CH_2Cl_2/MeOH 9:1).

^{1}H NMR: 300 MHz, DMSO-d_6; δ/ppm = 1.31 (d, 3H, ^3J = 7.1 Hz, Ala-CH_3), 4.15 – 4.30 (m, 4H, Ala-α-H, Fmoc-CH_2, Fmoc-CH), 7.27 (t, 1H, ^3J = 7.8 Hz, 5-H), 7.33 (pt, 2H, ^3J = 7.3 Hz, Fmoc-H_{ar}), 7.41 (dt, 2H, ^4J = 1.4 Hz, ^3J = 7.3 Hz, Fmoc-H_{ar}), 7.48 (dt, 1H, ^4J = 1.3 Hz, ^3J = 7.3 Hz, 6-H), 7.65 (d, 1H, ^3J = 7.4 Hz, Ala-NH), 7.69 – 7.77 (m, 3H, 4-H, Fmoc-H_{ar}), 7.85 – 7.91 (m, 3H, 2-H, Fmoc-H_{ar}), 8.06 (s, 2H, $B(OH)_2$), 9.91 (s, 1H, Ph-NH). **^{13}C NMR:** 75 MHz, DMSO-d_6; δ/ppm = 18.1 (Ala-CH_3), 46.7 (Fmoc-CH), 50.8 (Ala-α), 65.7 (Fmoc-CH_2), 120.1 (Fmoc-C_{ar}), 121.3 (C4), 125.3 (C2), 125.3 (Fmoc-C_{ar}), 127.1 (Fmoc-C_{ar}), 127.7 (Fmoc-C_{ar}), 127.7 (C5), 129.1 (C6), 138.2 (C3), 140.7 (Fmoc-$C_{ar,q}$), 143.9 (Fmoc-$C_{ar,q}$), 156.8 (Fmoc-C=O), 171.5 (Gly-C=O). **HR-ESI-MS:** calc. $[C_{24}H_{23}B_1N_2O_5Na_1]^+$: 453.1597, found: 453.1597. **IR:** ν/cm^{-1} = 3307 (w), 1671 (m), 1612 (w), 1585 (w), 1538 (m), 1488 (w), 1449 (m), 1428 (m), 1338 (s), 1251 (s), 1077 (m), 1033 (m), 798 (w), 759 (m), 739 (s), 708 (s), 665 (w), 520 (w), 426 (w).

H-Leu-Val-Phe-Phe-Ala-*m*PhB(OH)$_2$·TFA (1·TFA)

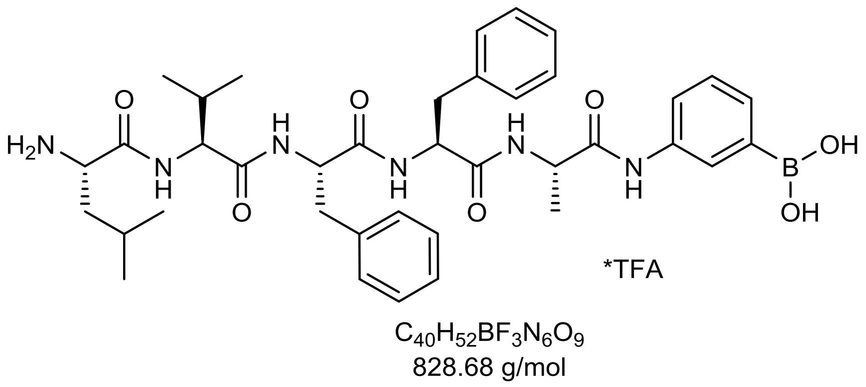

1

1 was obtained according to the solid-phase protocol on PS-DEAM (loading: 0.17 mmol/300 mg) with a yield of 64% (90 mg, 0.11 mmol).

^{1}H NMR: 500 MHz, DMSO-d_6; δ/ppm = 0.80 (d, 6H, ^3J = 6.8 Hz, 2x Val-CH_3), 0.81 (d, 3H, ^3J = 6.6 Hz, Leu-CH_3), 0.84 (d, 3H, ^3J = 6.6 Hz, Leu-CH_3), 1.32 (d, 3H, ^3J = 7.1 Hz, Ala-CH_3), 1.25 – 1.47 (m, 2H, Leu-β-H_2), 1.49 – 1.55 (m, 1H, Leu-γ-H), 1.85 – 1.92 (m, 1H, Val-β-H), 2.73 (dd, 1H, ^3J = 10.0 Hz, ^2J = 14.1 Hz, Phe^3-β-H^h), 2.83 (dd, 1H, ^3J = 8.9 Hz, ^2J = 13.9 Hz, Phe^4-β-H^h), 2.95 (dd, 1H, ^3J = 4.0 Hz, ^2J = 14.1 Hz, Phe^3-β-H^t), 3.07 (dd, 1H, ^3J = 4.4 Hz, ^2J = 13.9 Hz, Phe^4-β-H^t), 3.80 – 3.85 (m, 1H, Leu-α-H), 4.19 (dd, 1H, ^3J = 7.3 Hz, ^3J = 9.1 Hz, Val-α-H), 4.44 (m, 1H, ^3J = 7.2 Hz, Ala-α-H), 4.45 – 4.62 (m, 2H, Phe^3-α-H, Phe^4-α-H), 7.11 – 7.16 (m, 2H, Phe-H_{ar}), 7.17 – 7.26 (m, 8H, Phe-H_{ar}), 7.27 (t, 1H, ^3J = 7.7 Hz, 5-H), 7.49 (dt, 1H, ^4J = 1.3 Hz, ^3J = 7.3 Hz, 6-H), 7.71 (ddd, 1H, ^4J = 1.2 Hz, ^4J = 2.4 Hz, ^3J = 8.1 Hz, 4-H), 7.85 (s, 1H, 2-H), 8.03 (bs, 5H, $B(OH)_2$, Leu-ε-NH_3^+), 8.12 (d, 1H, ^3J = 8.2 Hz, Phe^4-NH), 8.14 (d, 1H, ^3J = 8.4 Hz, Phe^3-NH), 8.24 (d, 1H, ^3J = 7.3 Hz, Ala-NH), 8.35 (d, 1H, ^3J = 9.1 Hz, Val-NH), 9.88 (s, 1H, Ph-NH). **^{13}C NMR:** 125 MHz, DMSO-d_6; δ/ppm = 18.0 (Ala-CH_3), 18.0 (Val-CH_3), 18.8 (Val-CH_3), 21.6 (Leu-CH_3), 22.5 (Leu-CH_3), 23.2 (Leu-γ), 30.7 (Val-β), 37.1 (Phe-β), 37.2 (Phe-β), 40.1 (Leu-β), 48.8 (Ala-α), 50.4 (Leu-α), 53.1 (Phe-α), 53.3 (Phe-α), 57.3 (Val-α), 120.9 (C4), 124.9 (C2), 125.8 (Phe-C_{ar}), 125.8 (Phe-C_{ar}), 127.3 (C5), 127.6 (Phe-C_{ar}), 127.6 (Phe-C_{ar}), 127.6 (Phe-C_{ar}), 128.6 (Phe-C_{ar}), 128.8 (C6), 128.8 (Phe-C_{ar}). **HR-ESI-MS:** calc. $[C_{39}H_{53}B_1N_6O_7Na_1]^+$: 751.3968, found: 751.3954. (1x boronic acid methyl ester)

***m*PhB(OH)$_2$-Leu-Val-Phe-Phe-Ala-OH (2)**

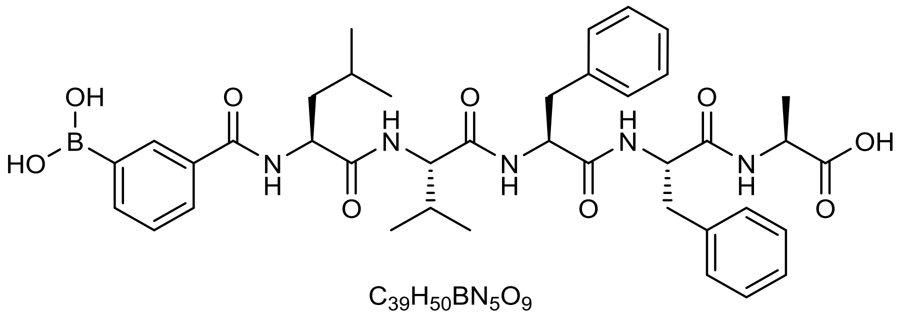

2

Peptideboronic acid **2** was synthesized on CTC-resin (loading: 0.18 mmol/250 mg) (Chlorotrityl Chloride-resin) according to the general protocol (108 mg, 0.15 mmol, 83%).

^{1}H NMR: 500 MHz, DMSO-d_6; δ/ppm = 0.70 (d, 6H, ^3J = 6.7 Hz, 2x Val-CH_3), 0.85 (d, 3H, ^3J = 6.4 Hz, Leu-CH_3), 0.90 (d, 3H, ^3J = 6.4 Hz, Leu-CH_3), 1.28 (d, 3H, ^3J = 7.3 Hz, Ala-CH_3), 1.43 – 1.49 (m, 1H, Leu-β-H^h), 1.63 – 1.71 (m, 2H, Leu-γ-H, Leu-β-H^t), 1.82 – 1.91 (m, 1H, Val-β-H), 2.72 (dd, 1H, ^3J = 9.1 Hz, ^2J = 14.1 Hz, Phe^3-β-H^h), 2.80 (dd, 1H, ^3J = 9.6 Hz, ^2J = 14.2 Hz, Phe^4-β-H^h), 2.92 (dd, 1H, ^3J = 4.6 Hz, ^2J = 14.1 Hz, Phe^3-β-H^t), 3.02 (dd, 1H, ^3J = 4.3 Hz, ^2J = 14.2 Hz, Phe^4-β-H^t), 4.12 (dd, 1H, ^3J = 6.8 Hz, ^3J = 8.8 Hz, Val-α-H), 4.21 (pquin, 1H, ^3J = 7.3 Hz, Ala-α-H), 4.47 – 4.57 (m, 3H, Leu-α-H, Phe^3-α-H, Phe^4-α-H), 7.11 – 7.20 (m, 6H, Phe-H_{ar}), 7.21 – 7.25 (m, 4H, Phe-H_{ar}), 7.43 (t, 1H, ^3J = 7.5 Hz, 5-H), 7.64 (d, 1H, ^3J = 9.0 Hz, Val-NH), 7.87 (dt, 1H, ^4J = 1.7 Hz, ^3J = 7.8 Hz, 4-H), 7.92 (dt, 1H, ^4J = 1.4 Hz, ^3J = 7.4 Hz, 6-H), 7.97 (d, 1H, ^3J = 8.5 Hz, Phe^3-NH), 8.00 (d, 1H, ^3J = 8.3 Hz, Phe^4-NH), 8.16 (bs, 2H, $B(OH)_2$), 8.24 (d, 1H, ^3J = 7.2 Hz, Ala-NH), 8.26 (s, 1H, 2-H), 8.40 (d, 1H, ^3J = 8.1 Hz, Leu-NH), 12.56 (bs, 1H, COOH). **^{13}C NMR:** 125 MHz, DMSO-d_6; δ/ppm = 17.0 (Ala-CH_3), 17.7 (Val-CH_3), 19.5 (Val-CH_3), 21.4 (Leu-CH_3), 23.0 (Leu-CH_3), 24.6 (Leu-γ), 30.5 (Val-β), 37.5 (Phe^3-β), 37.7 (Phe^4-β), 39.8 (Leu-β), 47.5 (Ala-α), 52.0 (Leu-α), 53.4 (Phe^4-α), 53.6 (Phe^3-α), 57.3 (Val-α), 126.1 (Phe-C_{ar}), 126.3 (Phe-C_{ar}), 127.3 (C5), 128.0 (Phe-C_{ar}), 128.0 (Phe-C_{ar}), 128.4 (Phe-C_{ar}), 128.9 (C4), 128.9 (Phe-C_{ar}), 129.1 (Phe-C_{ar}), 133.1 (C2), 136.8 (C6), 137.4 (Phe^4-$C_{ar,q}$), 137.5 (Phe^3-$C_{ar,q}$), 166.7 (Ph-C=O), 170.2 (Phe^4-C=O), 170.3 (Val-C=O), 170.4 (Phe^3-C=O), 172.0 (Leu-C=O), 173.7 (COOH). **HR-ESI-MS:** calc. $[C_{40}H_{50}B_1N_5O_9H_1]^-$: 756.3792, found: 756.3793 (1x boronic acid methyl ester).

8a(*S*)H-(*6S*)-Azido-(*7S*,*8S*)-hydroxy-5-oxo-hexahydrothiazolo[3,2-a]pyridine-3(*R*)-carboxylic acid methyl ester (5)

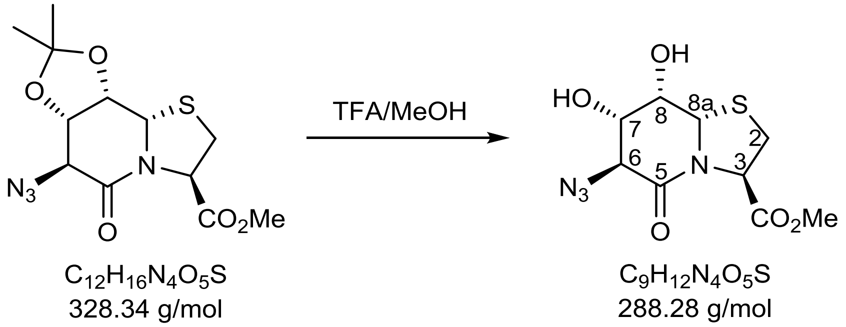

S2 **5**

The synthesis of S2 is described in Lit 24. Azide **S2** (100 mg, 0.31 mmol, 1.0 equiv) was dissolved in MeOH (2 mL) and treated with TFA (1 mL). After stirring for 3.5 h the solvent was removed under high vacuum and the residue was purified by flash chromatography (DCM/MeOH 20:1 → 9:1). Diol **5** (78 mg, 0.27 mmol, 89%) was obtained as a colourless solid. **Tlc:** R_f = 0.55 (DCM/MeOH 9:1).

^{1}H NMR: 500 MHz, DMSO-d_6; δ/ppm = 3.06 (dd, 1H, ^3J = 5.4 Hz, ^2J = 11.4 Hz, 2-H^h), 3.29 (dd, 1H, ^3J = 7.2 Hz, ^2J = 11.4 Hz, 2-H^t), 3.69 (s, 3H, CO_2CH_3), 3.72 (ddd, 1H, ^3J = 1.9 Hz, ^3J = 6.2 Hz, ^3J = 9.8 Hz, 7-H), 3.95 (pdt, 1H, ^3J = 2.0 Hz, ^3J = 5.2 Hz, 8-H), 4.13 (d, 1H, ^3J = 9.9 Hz, 6-H), 4.98 (d, 1H, ^3J = 1.9 Hz, 8a-H), 4.99 (dd, 1H, ^3J = 5.7 Hz, ^3J = 7.4 Hz, 3-H), 5.70 (d, 1H, ^3J = 5.3 Hz, 8-OH), 5.70 (d, 1H, ^3J = 6.1 Hz, 7-OH). **^{13}C NMR:** 125 MHz, DMSO-d_6; δ/ppm = 30.8 (C2), 52.5 (CO_2CH_3), 61.0 (C3), 62.7 (C6), 64.9 (C8a), 67.8 (C8), 70.6 (C7), 164.7 (C5), 169.8 (CO_2Me). **HR-ESI-MS:** calc. $[C_9H_{12}N_4O_5S_1Na_1]^+$: 311.0421, found: 311.0418. **IR:** ν/cm^{-1} = 3430 (m), 2955 (w), 2110 (s), 1736 (m), 1643 (s), 1427 (m), 1353 (w), 1250 (m), 1213 (s), 1144 (m), 1110 (m), 1050 (w), 1013 (m), 929 (w), 826 (w), 799 (w), 761 (w), 622 (w), 595 (w), 551 (w).

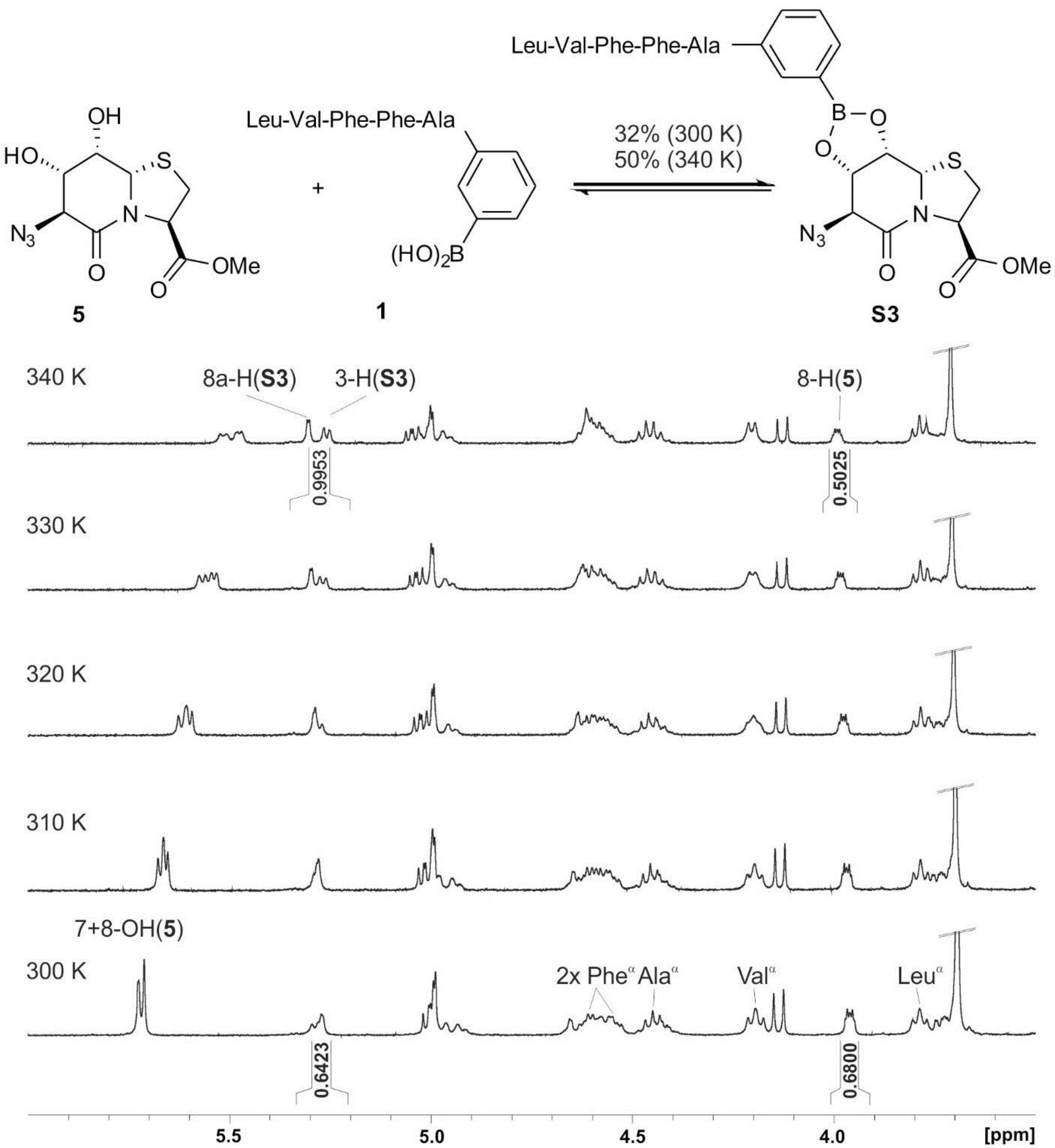

Figure S2: Expansions from ^{1}H NMR spectra (400 MHz, DMSO-d_6) between 300 and 340 K. The boronic ester formation between diol **5** and peptide boronic acid **1** to **S3** increases with the temperature. Over the temperature range of 40 K the conversion rises by 18%. These data were obtained from the ratio of the integrals of educt **5** (8-H, 3.95 ppm) and product **S3** (8a-H,3-H, 5.2-5.3 ppm).

Azido-(Hot=Tap)$_2$-OMe (9)

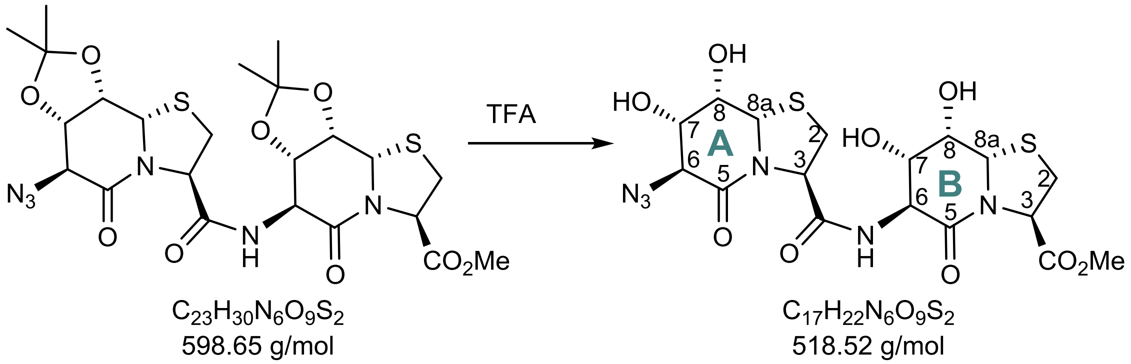

S4 **9**

Dimer **S4** (100 mg, 0.17 mmol, 1.0 equiv) was suspended in DCM (5 mL) and treated with TFA (8 mL). The solution was stirred for 4 h before the solvent was removed under reduced pressure. Reaction control by ^{1}H NMR identified traces amounts of remaining isopropylidene protecting groups and the residue was stirred a second time in DCM/TFA (8 h). After removing the solvent, the product was taken up in TFA (0.5 mL) and precipitated from cold Et_2O_{abs}. Centrifugation and washing yielded tetraol **9** (79 mg, 0.15 mmol, 90%) as a colourless solid.

^{1}H NMR: 500 MHz, DMSO-*d$_6$*; δ/ppm = 3.02 (dd, 1H, ^3J = 4.4 Hz, ^2J = 11.3 Hz, 2^B-H^h), 3.07 (dd, 1H, ^3J = 4.5 Hz, ^2J = 11.1 Hz, 2^A-H^h), 3.14 (dd, 1H, ^3J = 7.3 Hz, ^2J = 11.1 Hz, 2^A-H^t), 3.23 (dd, 1H, ^3J = 7.2 Hz, ^2J = 11.3 Hz, 2^B-H^t), 3.66 (s, 3H, CO_2CH_3), 3.76 (dd, 1H, ^3J = 1.9 Hz, ^3J = 10.0 Hz, 7^A-H), 3.92 (pt, 1H, ^3J = 2.1 Hz, 8^A-H), 3.96 (dd, 1H, ^3J = 2.1 Hz, ^3J = 10.0 Hz, 7^B-H), 3.99 (pt, 1H, ^3J = 2.1 Hz, 8^B-H), 4.13 (d, 1H, ^3J = 10.0 Hz, 6^A-H), 4.33 (dd, 1H, ^3J = 8.9 Hz, ^3J = 10.0 Hz, 6^B-H), 4.97 – 5.02 (m, 4H, 8a^A-H, 8a^B-H, 3^A-H, 3^B-H), 5.58 (bs, 4H, 7^A-OH, 7^B-OH, 8^A-OH, 8^B-OH), 8.34 (d, 1H, ^3J = 8.9 Hz, NH^B). **^{13}C NMR:** 125 MHz, DMSO-*d$_6$*; δ/ppm = 30.9 (C2^B), 31.6 (C2^A), 52.2 (C6^B), 52.3 (CO_2CH_3), 61.1 (C3^B), 61.8 (C3^A), 62.8 (C6^A), 64.4 (C8a^B), 65.3 (C8a^A), 67.9 (C8^B), 68.1 (C8^A), 69.4 (C7^B), 70.3 (C7^A), 164.8 (C5^A), 165.9 (C5^B), 169.4 (C=O^A), 170.2 (CO_2Me). **HR-ESI-MS:** calc. $[C_{17}H_{22}N_6O_9S_2Na_1]^+$: 541.0782, found: 541.0776.

Azido-{Hot=Tap[(*7S,8S*)- *meta*-[*N*-(Acetyl)amino]-phenyl-[1,3,2]dioxaborolo]}$_2$-OMe (9) and 6 in NMR titration: (compounds 10, 11, and 12 in Table 1)

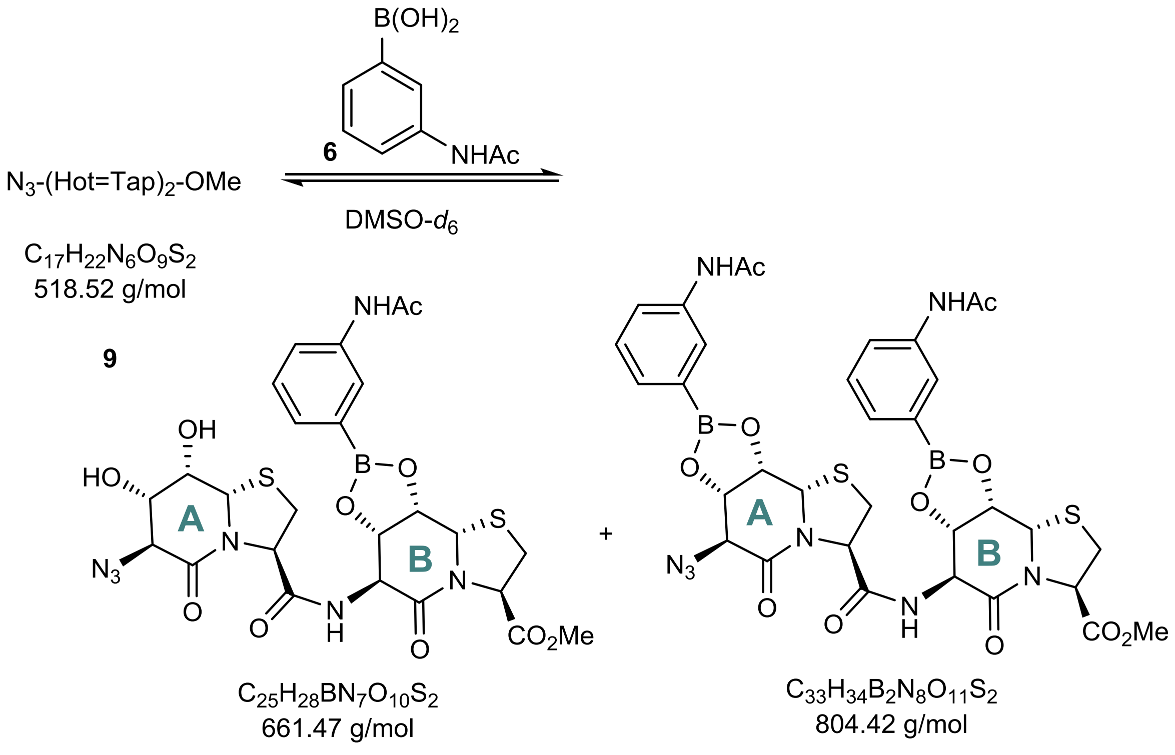

10, 11 **12**

Tetraol **9** (6.90 mg, 13.3 µmol, 1.0 equiv) was dissolved in an NMR tube DMSO-d_6 (0.7 mL) together with 3-(acetylamino)phenylboronic acid **6** (3.10 mg, 17.3 µmol, 1.3 equiv). 58% conversion (4.5 equiv H_2O) relative to boronic acid **6** was observed with a ratio of **9/10/11/12** listed in Table 1 (I). The solvent was dried over molecular sieves to increase the conversion to 90% (0.8 equiv H_2O) with a ratio listed in Table 1 (II). Then **6** (1.67 mg, 9.36 µmol, 0.7 equiv) was added again and the solvent dried over molecular sieves. The conversion was >90% (1.3 equiv H_2O) with the ratio given in Table 1 (III).

Compound 11: **^{1}H NMR:** 500 MHz, DMSO-d_6; δ/ppm = 2.03 (s, 3H, Ac-CH$_3$), 2.93 (dd, 1H, ^3J = 6.4 Hz, ^2J = 11.5 Hz, 2^A-H^h), 3.05 – 3.10 (m, 1H, 2^B-H^h), 3.19 – 3.23 (m, 1H, 2^B-H^t), 3.35 (dd, 1H, ^3J = 7.7 Hz, ^2J = 11.5 Hz, 2^A-H^t), 3.67 (s, 3H, CO$_2$CH$_3$), 3.70 – 3.74 (m, 1H, 7^A-H), 3.98 – 4.00 (m, 1H, 8^A-H), 4.15 (d, 1H, ^3J = 9.8 Hz, 6^A-H), 4.49 (dd, 1H, ^3J = 1.8 Hz, ^3J = 7.3 Hz, 6^B-H), 4.88 – 4.90 (m, 1H, 3^A-H), 4.95 – 5.03 (m, 3H, 8a^A-H, 8^B-H, 7^B-H), 5.26 (dd, 1H, ^3J = 1.4 Hz, ^3J = 6.7 Hz, 3^B-H), 5.41 (s, 1H, ^3J = 2.1 Hz, 8a^B-H), 5.68 (d, 1H, ^3J = 4.3 Hz, 7^A-OH), 5.70 (d, 1H, ^3J = 6.0 Hz, 8^A-OH), 7.31 – 7.34 (m, 2H, 5'-H, 6'-H), 7.79 – 7.84 (m, 2H, 2'-H, 4'-H), 8.79 (d, 1H, ^3J = 7.2 Hz, NH^B), 9.98 (s, 1H, NHAc).

Compound 12: **^{1}H NMR:** 500 MHz, DMSO-d_6; δ/ppm = 2.02 (s, 3H, Ac-CH$_3$), 2.04 (s, 3H, Ac-CH$_3$), 3.05 (dd, 1H, ^3J = 1.0 Hz, ^2J = 11.4 Hz, 2^A-H^h), 3.08 (dd, 1H, ^3J = 6.4 Hz, ^2J = 11.5 Hz, 2^B-H^h), 3.19 – 3.23 (m, 2H, 2^A-H^t, 2^B-H^t), 3.68 (s, 3H, CO$_2$CH$_3$), 4.51 (dd, 1H, ^3J = 1.9 Hz, ^3J = 7.8 Hz, 6^B-H), 4.66 (d, 1H, ^3J = 1.9 Hz, 6^A-H), 4.88

(dd, 1H, ^3J = 1.9 Hz, ^3J = 7.7 Hz, 7^B-H), 4.96 – 5.00 (m, 3H, 8^A-H, 8^B-H, 7^A-H), 5.02 (dd, 1H, ^3J = 1.8 Hz, ^3J = 6.4 Hz, 3^A-H), 5.26 (dd, 1H, ^3J = 1.0 Hz, ^3J = 6.3 Hz, 3^B-H), 5.35 (s, 1H, ^3J = 1.3 Hz, 8a^A-H), 5.37 (s, 1H, ^3J = 2.0 Hz, 8a^B-H), 7.30 – 7.35 (m, 2H, 5'-H, 6'-H), 7.79 – 7.88 (m, 2H, 2'-H, 4'-H), 8.59 (d, 1H, ^3J = 7.8 Hz, NH^B), 9.97 (s, 1H, NHAc), 9.98 (s, 1H, NHAc).

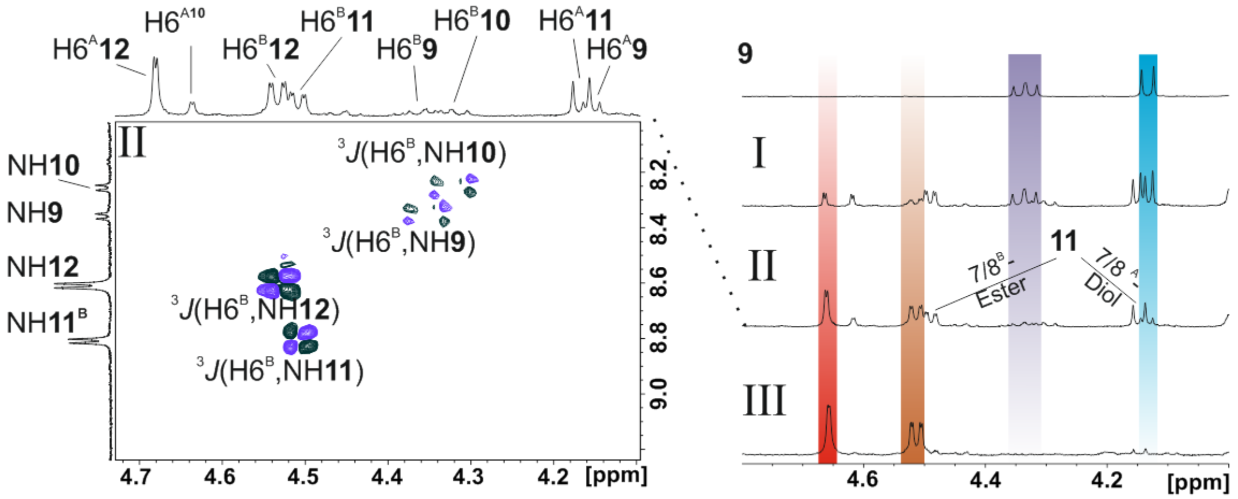

Figure S3: *Right*: ^{1}H NMR titration (500 MHz, DMSO-d_6, 300 K) of tetraol **9** with boronic acid **6**. The expansion shows the region of 6-H of Hot. The two bars at higher field show the decrease of 6-H^A (light blue) and 6-H^B (purple) of Hot diols. The other two bars show the increase of the esterified Hot 6-H^A (red) and 6-H^B (brown). *Left:* Expansion from the DQF-COSY spectrum at ratio II. The four amide NH are clearly separated.

Azido-Hot=Tap[(*7S,8S*)-*meta*-[*N*-(acetyl)amino]phenyl[1,3,2]dioxaborolo]-OMe (5), 2-formylphenyl-boronic acid (14) and peptide LVFFA in NMR titration: (15)

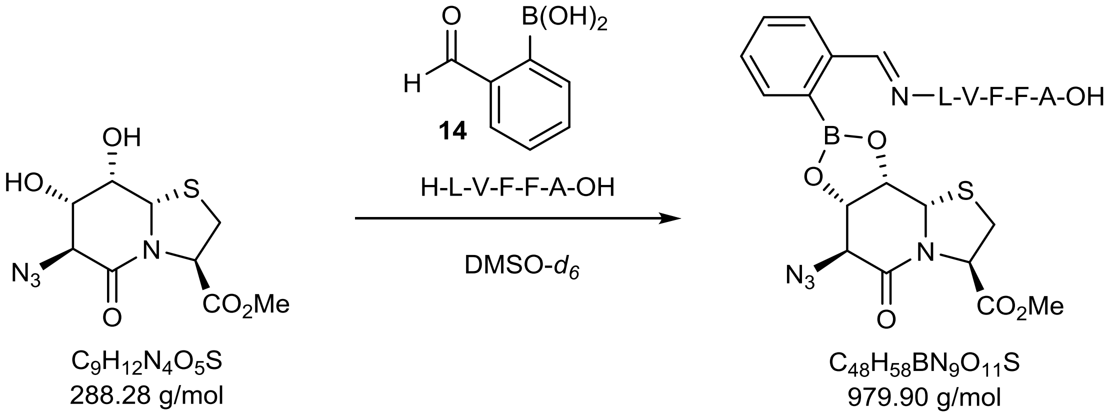

5 **15**

2-Formylphenylboronic acid (1.49 mg, 9.94 µmol, 1.0 equiv) and peptide LVFFA (5.95 mg, 9.98 µmol, 1.0 equiv) were dissolved in 0.7 mL DMSO-d_6 and treated with diol **5** (2.88 mg, 9.99 µmol, 1.0 equiv). Conversion was quantitative in NMR to the iminoboronate ester **15** (11.2 equiv H_2O).

^{1}H NMR: 500 MHz, DMSO-d_6; δ/ppm = 0.74 (d, 3H, ^3J = 6.8 Hz, Val-CH_3), 0.76 (d, 3H, ^3J = 6.80 Hz, Val-CH_3), 0.86 (d, 3H, ^3J = 6.3 Hz, Leu-CH_3), 0.90 (d, 3H, ^3J = 6.3 Hz, Leu-CH_3), 1.28 (d, 3H, ^3J = 7.2 Hz, Ala-CH_3), 1.35 – 1.49 (m, 2H, Leu-β-H^h, Leu-γ-H), 1.81 – 1.90 (m, 2H, Leu-β-H^t, Val-β-H), 2.71 (dd, 1H, ^3J = 9.9 Hz, ^2J = 14.3 Hz, Phe^3-β-H^h), 2.81 (dd, 1H, ^3J = 9.0 Hz, ^2J = 13.8 Hz, Phe^4-β-H^h), 2.93 (dd, 1H, ^3J = 4.2 Hz, ^2J = 14.3 Hz, Phe^3-β-H^t), 3.00 (dd, 1H, ^3J = 6.3 Hz, ^2J = 11.4 Hz, 2-H^h), 3.04 (dd, 1H, ^3J = 4.4 Hz, ^2J = 13.8 Hz, Phe^4-β-H^t), 3.09 (dd, 1H, ^3J = 1.3 Hz, ^2J = 11.4 Hz, 2-H^t), 3.70 (s, 3H, CO_2CH_3), 4.14 (d, 1H, ^3J = 2.2 Hz, 6-H), 4.16 (dd, 1H, ^3J = 6.9 Hz, ^3J = 9.3 Hz, Val-α-H), 4.22 (pquin, 1H, ^3J = 7.2 Hz, Ala-α-H), 4.52 (ddd, 1H, ^3J = 4.0 Hz, ^3J = 8.3 Hz, ^3J = 9.9 Hz, Phe^4-α-H), 4.56 – 4.61 (m, 2H, Phe^3-α-H, Leu-α-H), 4.68 (dd, 1H, ^3J = 1.8 Hz, ^3J = 7.1 Hz, 8-H), 4.72 (dd, 1H, ^3J = 2.2 Hz, ^3J = 7.1 Hz, 7-H), 5.15 (d, 1H, ^3J = 1.9 Hz, 8a-H), 5.44 (dd, 1H, ^3J = 1.3 Hz, ^3J = 6.1 Hz, 3-H), 7.03 (d, 1H, ^3J = 7.0 Hz, 6'-H), 7.15 – 7.27 (m, 10H, Phe-H_{ar}), 7.30 (dt, 1H, ^4J = 1.2 Hz, ^3J = 7.5 Hz, 4'-H), 7.42 (dt, 1H, ^4J = 1.2 Hz, ^3J = 7.4 Hz, 5'-H), 7.63 (d, 1H, ^4J = 7.4 Hz, 3'-H), 8.02 (d, 1H, ^3J = 8.3 Hz, Phe^4-NH), 8.16 (d, 1H, ^3J = 8.4 Hz, Phe^3-NH), 8.30 (d, 1H, ^3J = 7.2 Hz, Ala-NH), 8.51 (d, 1H, ^3J = 9.3 Hz, Val-NH), 8.86 (s, 1H, N=CH), 12.55 (bs, 1H, COOH). **^{13}C-NMR:** 125 MHz, DMSO-d_6; δ/ppm = 17.1 (Ala-CH_3), 17.8 (Val-CH_3), 19.1 (Val-CH_3), 21.0 (Leu-CH_3), 23.1 (Leu-CH_3), 23.9 (Leu-γ), 31.2 (Val-β), 31.3 (C2), 37.5 (Phe^4-β), 37.6 (Phe^3-β), 40.7 (Leu-β), 47.4 (Ala-α), 52.6 (CO_2CH_3), 53.3 (Phe^4-α), 53.5 (Phe^3-α), 57.7 (Val-α), 57.8 (Leu-α), 61.3 (C3), 62.0 (C8a), 63.8 (C6), 74.4 (C7), 75.7 (C8), 126.0 (Phe-C_{ar}), 126.1 (Phe-C_{ar}), 126.8 (C3'), 127.9 (Phe-C_{ar}), 127.9 (Phe-C_{ar}), 128.0 (C4'), 129.0 (Phe-C_{ar}), 129.0 (Phe-C_{ar}), 129.1 (C6'), 132.7 (C5'), 137.4 (Phe-$C_{ar,q}$), 137.7 (Phe-$C_{ar,q}$), 138.3 (C2'), 164.1 (C5), 168.3 (Leu-C=O), 168.9 (N=CH), 169.4 (CO_2Me), 170.3 (Val-C=O), 170.5 (Phe^4-C=O), 170.7 (Phe^3-C=O), 173.8 (COOH).

Azido-{Hot=Tap[(*7S,8S*)-*meta*-[*N*-(acetyl)amino]-phenyl-[1,3,2]dioxaborolo]}$_2$-OMe (9) and 2-formylphenylboronic acid (14) and peptide LVFFA in NMR titration: (S5)

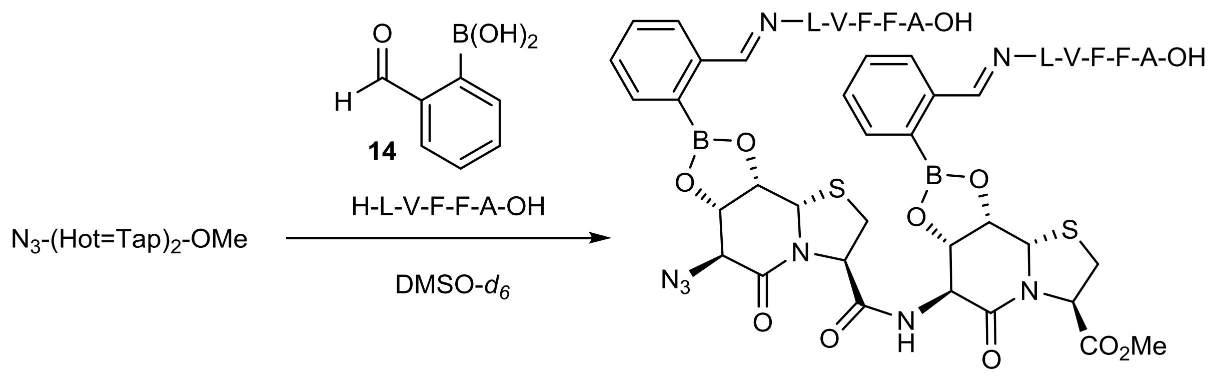

$C_{17}H_{22}N_6O_9S_2$
518.52 g/mol

9

$C_{95}H_{114}B_2N_{16}O_{21}S_2$
1901.77 g/mol

S5

2-Formylphenylboronic acid (2.30 mg, 15.3 µmol, 2.0 equiv) and peptide LVFFA (9.18 mg, 15.4 µmol, 2.0 equiv) were dissolved in 0.7 mL DMSO-d_6 and treated with tetraol **9** (4.00 mg, 7.71 µmol, 1.0 equiv). Conversion was quantitative in NMR to the double iminoboronate ester **S5** (31.0 equiv H_2O).

^{1}H NMR: 500 MHz, DMSO-*d_6*; δ/ppm = 0.69 – 0.79 (m, 12H, Val^A-CH_3, Val^B-CH_3), 0.87 (d, 3H, ^3J = 6.2 Hz, Leu$^{A/B}$-$CH_3{}^h$), 0.87 (d, 3H, ^3J = 6.3 Hz, Leu$^{A/B}$-$CH_3{}^h$), 0.90 (d, 3H, ^3J = 6.3 Hz, Leu$^{A/B}$-$CH_3{}^t$), 0.91 (d, 3H, ^3J = 6.4 Hz, Leu$^{A/B}$-$CH_3{}^t$), 1.28 (d, 3H, ^3J = 7.3 Hz, Ala$^{A/B}$-CH_3), 1.29 (d, 3H, ^3J = 7.3 Hz, Ala$^{A/B}$-CH_3), 1.36 – 1.49 (m, 4H, Leu^A-β-H^h, Leu^B-β-H^h, Leu^A-γ-H, Leu^B-γ-H), 1.79 – 1.91 (m, 4H, Leu^A-β-H^t, Leu^B-β-H^t, Val^A-β-H, Val^B-β-H), 2.69 – 2.74 (m, 2H, Phe^{3A}-β-H^h, Phe^{3B}-β-H^h), 2.78 – 2.85 (m, 3H, 2^A-H^h, Phe^{4A}-β-H^h, Phe^{4B}-β-H^h), 2.91 (dd, 1H, ^3J = 3.9 Hz, ^2J = 14.1 Hz, Phe$^{3A/B}$-β-H^t), 2.91 (dd, 1H, ^3J = 3.7 Hz, ^2J = 14.1 Hz, Phe$^{3A/B}$-β-H^t), 3.00 – 3.12 (m, 5H, Phe^{4A}-β-H^t, Phe^{4B}-β-H^t, 2^A-H^t, 2^B-H_2), 3.68 (s, 3H, CO_2CH_3), 4.07 (d, 1H, ^3J = 2.2 Hz, 6^A-H), 4.09 (dd, 1H, ^3J = 7.1 Hz, ^3J = 8.6 Hz, Val^B-α-H), 4.14 – 4.18 (m, 1H, Val^A-α-H), 4.21 (pquin, 1H, ^3J = 7.3 Hz, Ala$^{A/B}$-α-H), 4.22 (pquin, 1H, ^3J = 7.3 Hz, Ala$^{A/B}$-α-H), 4.46 (dd, 1H, ^3J = 1.9 Hz, ^3J = 9.3 Hz, 6^B-H), 4.49 – 4.63 (m, 7H, Phe^{3A}-α-H, Phe^{3B}-α-H, Phe^{4A}-α-H, Phe^{4B}-α-H, Leu^A-α-H, Leu^B-α-H, 7^B-H), 4.67 (dd, 1H, ^3J = 1.9 Hz, ^3J = 7.4 Hz, 8^A-H), 4.72 (dd, 1H, ^3J = 2.2 Hz, ^3J = 7.1 Hz, 8^B-H), 4.74 (dd, 1H, ^3J = 1.9 Hz, ^3J = 7.2 Hz, 7^A-H), 5.17 (d, 1H, ^3J = 1.9 Hz, 8a^A-H), 5.35 (pd, 1H, ^3J = 6.4 Hz, 3^A-H), 5.38 – 4.11 (m, 2H, 3^B-H, 8a^B-H), 7.03 (d, 1H, ^3J = 7.0 Hz, 6$^{A'}$-H), 7.07 (d, 1H, ^3J = 7.0 Hz, 6$^{B'}$-H), 7.16 – 7.26 (m, 20H, Phe^A-H_{ar}, Phe^B-H_{ar}), 7.31 (dt, 1H, ^4J = 1.2 Hz, ^3J = 7.5 Hz, 4$^{B'}$-H), 7.34 (dt, 1H, ^4J = 1.2 Hz, ^3J = 7.5 Hz, 4$^{A'}$-H), 7.41 – 7.46 (m, 2H, 5$^{A'}$-H, 5$^{B'}$-H), 7.63 (d, 1H, ^4J = 7.6 Hz, 3$^{B'}$-H), 7.66 (d, 1H, ^4J = 7.4 Hz, 3$^{A'}$-H), 8.01 – 8.17 (m, 5H, Phe^{3A}-NH, Phe^{3B}-NH, Phe^{4A}-NH, Phe^{4B}-NH, 6^B-NH), 8.27 (d, 1H, ^3J = 7.1 Hz, Ala$^{A/B}$-NH), 8.29 (d, 1H, ^3J = 7.4 Hz, Ala$^{A/B}$-NH), 8.47 (d, 1H, ^3J = 9.2 Hz, Val^B-NH), 8.53 (d, 1H, ^3J = 8.9 Hz, Val^A-NH), 8.85 (s, 1H, N=CH^B), 8.87 (s, 1H, N=CH^A).
^{13}C-NMR: 125 MHz, DMSO-*d_6*; δ/ppm = 17.0 (Ala$^{A/B}$-CH_3), 17.6 (Val-CH_3), 17.7 (Val-CH_3), 18.8 (Val-CH_3), 18.9 (Val-CH_3), 20.9 (Leu$^{A/B}$-CH_3), 22.9 (Leu$^{A/B}$-CH_3), 23.7 (Leu-γ), 30.8 (Val$^{A/B}$-β), 30.9 (C2^B), 31.0 (Val$^{A/B}$-β), 31.8 (C2^A), 37.3 (Phe$^{3/4A}$-β), 37.4 (Phe$^{3/4B}$-β), 40.6 (Leu$^{A/B}$-β), 47.3 (Ala$^{A/B}$-α), 52.4 (CO_2*CH_3*), 53.2 (Phe$^{3/4,A/B}$-α), 53.5 (Phe$^{3/4,A/B}$-α), 54.8 (C6^B), 57.4 (Val^A-α), 57.7 (Leu$^{A/B}$-α), 57.9 (Val^B-α), 61.2 (C3^B), 61.3 (C8a^B), 61.5 (C3^A), 62.5 (C8a^A), 63.9 (C6^A), 74.4 (C7^A), 75.6 (C8^A), 75.9 (C8^B), 76.2 (C7^B), 125.8 (Phe-C_{ar}), 125.9 (Phe-C_{ar}), 126.5 (C3$^{A/B'}$), 127.4 (C4$^{A/B'}$), 127.5 (Phe-C_{ar}), 127.6 (Phe-C_{ar}), 128.5 (Phe-C_{ar}), 128.6 (C6$^{B'}$), 128.7 (C6$^{A'}$), 128.7 (Phe-C_{ar}), 128.8 (Phe-C_{ar}), 132.4 (C5$^{A/B'}$), 137.3 (Phe-$C_{ar,q}$), 137.6 (Phe-$C_{ar,q}$), 138.2 (C2$^{B'}$), 138.5 (C2$^{A'}$), 149.8 (C1$^{A/B'}$), 163.8 (C5^A), 166.2 (C5^B), 167.8 (Leu^B-C=O), 168.0 (Leu^A-C=O), 168.7 (N=CH$^{A/B}$), 169.7 (CO_2Me), 170.2 (3^A-C=O), 170.4 (Val$^{A/B}$-C=O), 170.5 (Phe$^{4A/B}$-C=O), 170.7 (Phe$^{3A/B}$-C=O), 173.8 (COOH).

Azido-(Hot=Tap)$_3$-OMe (16)

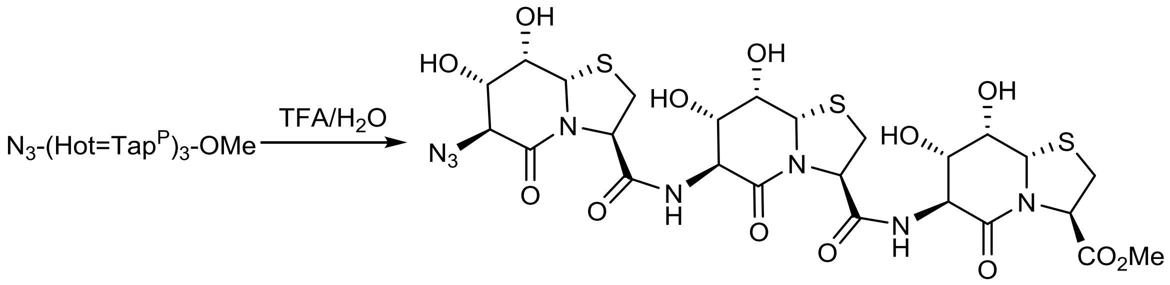

$C_{34}H_{44}N_8O_{13}S_3$
868.95 g/mol

S6

$C_{25}H_{32}N_8O_{13}S_3$
748.76 g/mol

16

Note: trimer **16** was synthesized according to dimer **9**. Deprotection: Trimer **S6** (70 mg, 80.6 µmol, 1.0 equiv) was dissolved in TFA (3 mL) and H_2O (0.2 mL) and stirred for 3.5 h. The residue was concentrated to 1 mL and the product was precipitated from cold Et_2O_{abs}. The precipitate was centrifugated and washed with Et_2O (2×) and dried under high vacuum. Hexaol **16** (53 mg, 70.8 µmol, 88%) was obtaind as a colourless solid. **Tlc:** R_f = 0.05 (DCM/MeOH 9:1).

^{1}H NMR: 600 MHz, DMSO-*d$_6$*; δ/ppm = 2.99 – 3.04 (m, 3H, 2^A-H^h, 2^B-H^h, 2^C-H^h), 3.09 (dd, 1H, ^3J = 7.2 Hz, ^2J = 11.3 Hz, 2^A-H^t), 3.21 – 3.24 (m, 2H, 2^B-H^t, 2^C-H^t), 3.66 (s, 3H, CO_2CH_3), 3.83 (dd, 1H, ^3J = 7.7 Hz, ^3J = 9.7 Hz, 6^B-H), 3.86 (ddd, 1H, ^3J = 2.0 Hz, ^3J = 6.4 Hz, ^3J = 10.0 Hz, 7^A-H), 3.90 (pdt, 1H, ^3J = 2.1 Hz, ^3J = 4.8 Hz, 8^C-H), 3.94 (pdt, 1H, ^3J = 2.1 Hz, ^3J = 5.0 Hz, 8^B-H), 3.96 (ddd, 1H, ^3J = 2.1 Hz, ^3J = 5.9 Hz, ^3J = 9.6 Hz, 7^B-H), 3.98 – 4.02 (m, 2H, 7^C-H, 8^A-H), 4.13 (d, 1H, ^3J = 9.9 Hz, 6^A-H), 4.38 (dd, 1H, ^3J = 9.2 Hz, ^3J = 10.4 Hz, 6^C-H), 4.88 (dd, 1H, ^3J = 4.9 Hz, ^3J = 7.0 Hz, 3^A-H), 4.98 – 5.00 (m, 3H, $8a^A$-H, $8a^B$-H, 3^C-H), 5.03 (d, 1H, ^3J = 1.7 Hz, $8a^C$-H), 5.12 (d, 1H, ^3J = 6.0 Hz, 7^C-OH), 5.17 (dd, 1H, ^3J = 2.6 Hz, ^3J = 6.9 Hz, 3^B-H), 5.23 (d, 1H, ^3J = 5.7 Hz, 7^B-OH), 5.45 (d, 1H, ^3J = 4.6 Hz, 8^C-OH), 5.51 (d, 1H, ^3J = 5.0 Hz, 8^A-OH), 5.61 (d, 1H, ^3J = 4.7 Hz, 8^B-OH), 5.61 (d, 1H, ^3J = 6.4 Hz, 7^A-H), 7.90 (d, 1H, ^3J = 9.0 Hz, NH^C), 8.81 (d, 1H, ^3J = 7.7 Hz, NH^B). **^{13}C NMR:** 125 MHz, DMSO-*d$_6$*; δ/ppm = 30.1 ($C2^B$), 30.9 ($C2^C$), 31.4 ($C2^A$), 52.3 ($C6^C$), 52.3 (CO_2CH_3), 53.9 ($C6^B$), 60.8 ($C3^C$), 61.4 ($C3^B$), 62.6 ($C3^A$), 62.8 ($C6^A$), 63.6 ($C8a^C$), 64.5 ($C8a^A$), 65.2 ($C8a^B$), 68.0 ($C8^A$), 68.1 ($C8^B$), 68.2 ($C8^C$), 69.5 ($C7^C$), 69.7 ($C7^B$), 70.0 ($C7^A$), 165.4 ($C5^A$), 165.6 ($C5^B$, $C5^C$), 169.2 ($C{=}O^B$), 170.1 ($C{=}O^A$), 170.2 (CO_2Me). **HR-ESI-MS:** calc. $[C_{25}H_{32}N_8O_{13}S_3Na_1]^+$: 771.1143, found: 771.1134.

Azido-{Hot=Tap[(*7S,8S*)-*meta*-[*N*-(acetyl)amino]phenyl[1,3,2]dioxaborolo]}$_3$-OMe (16) and 2-formylphenylboronic acid (14) and peptide LVFFA in NMR titration: (17)

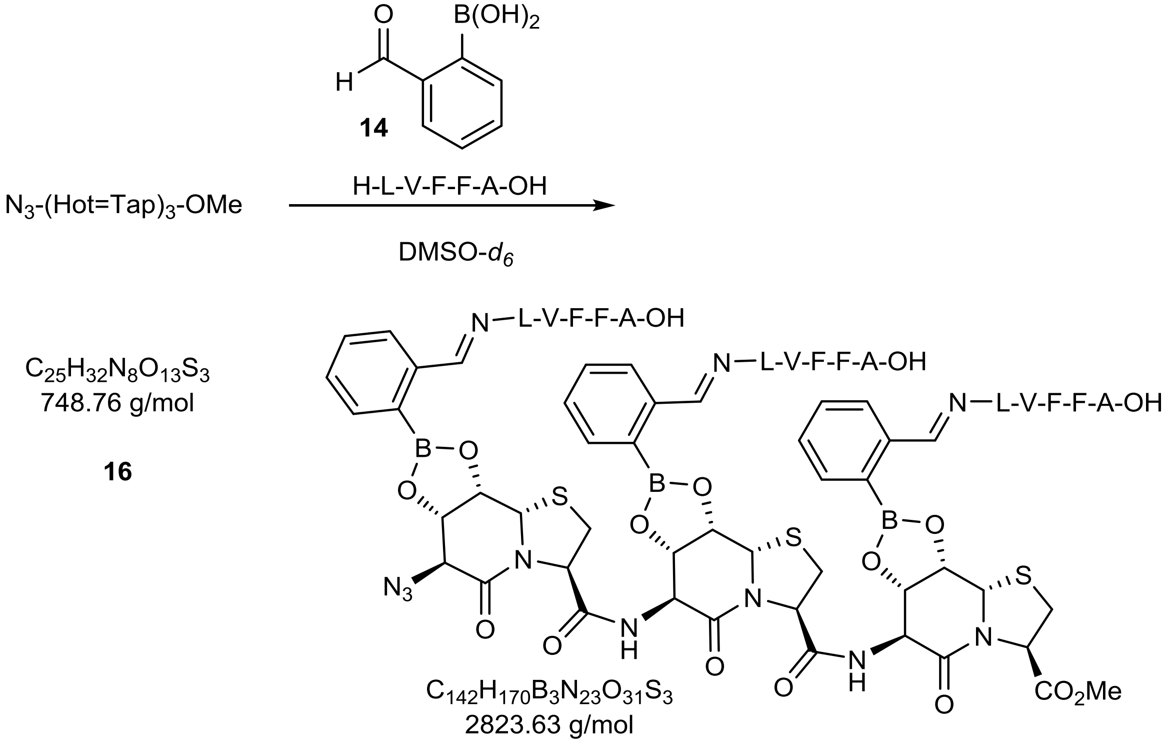

17

2-Formylphenylboronic acid (2.99 mg, 19.9 μmol, 3.0 equiv) and peptide LVFFA (11.9 mg, 19.9 μmol, 3.0 equiv) were dissolved in 0.7 mL DMSO-d_6 and treated with hexaol **16** (5.00 mg, 6.68 μmol, 1.0 equiv). Conversion was quantitative in NMR to the double iminoboronate ester **17** (31.0 equiv H_2O).

^{1}H NMR: 600 MHz, DMccSO-d_6; δ/ppm = 0.68 – 0.79 (m, 18H, Val^{A}-CH_3, Val^{B}-CH_3, Val^{C}-CH_3), 0.84 – 0.94 (m, 18H, Leu^{A}-CH_3, Leu^{B}-CH_3 Leu^{C}-CH_3), 1.26 – 1.31 (m, 9H, Ala^{A}-CH_3, Ala^{B}-CH_3, Ala^{C}-CH_3), 1.38 – 1.50 (m, 5H, Leu^{A}-γ-H^{h}, Leu^{B}-γ-H^{h}, Leu^{C}-γ-H^{h}, 2x Leu-β-H^{h}), 1.61 – 1.66 (m, 1H, Leu-β-H^{t}), 1.79 – 1.91 (m, 5H, 2x Leu-β-H^{t}, Val^{A}-β-H, Val^{B}-β-H, Val^{C}-β-H), 2.70 – 2.77 (m, 3H, Phe^{3A}-β-H^{h}, Phe^{3B}-β-H^{h}, Phe^{3C}-β-H^{h}), 2.79 – 2.85 (m, 3H, Phe^{4A}-H^{h}, Phe^{4B}-β-H^{h}, Phe^{4C}-β-H^{h}), 2.89 – 2.96 (m, 4H, Phe^{3A}-β-H^{t}, Phe^{3B}-β-H^{t}, Phe^{3C}-β-H^{t}, 2^{A}-H^{h}), 2.99 – 3.14 (m, 8H, Phe^{4A}-β-H^{t}, Phe^{4B}-β-H^{t}, Phe^{4C}-β-H^{t}, 2^{A}-H^{t}, 2^{B}-H_2, 2^{C}-H_2), 3.68 (s, 3H, CO_2CH_3), 4.05 – 4.11 (m, 3H, 6^{A}-H, 2x Val-α-H), 4.13 – 4.22 (m, 6H, Val-α-H, Ala^{A}-α-H, Ala^{B}-α-H, Ala^{C}-α-H, 6$^{B/C}$-H), 4.44 – 4.65 (m, 12H, 6$^{C/B}$-H, Phe^{3A}-α-H, Phe^{3B}-α-H, Phe^{3C}-α-H, Phe^{4A}-α-H, Phe^{4A}-α-H, Phe^{4A}-α-H, Leu^{A}-α-H, Leu^{B}-α-H, Leu^{C}-α-H, 2x 7-H), 4.66 – 4.78 (m, 4H, 7-H, 8^{A}-H, 8^{B}-H, 8^{C}-H), 5.19 (ps, 1H, 8a^{A}-H), 5.32 – 5.39 (m, 3H, 8a$^{B/C}$-H, 3^{A}-H, 3$^{B/C}$-H), 5.41 – 5.43 (m, 1H, 3$^{C/B}$-H), 5.45 – 5.47 (m, 1H, 8a$^{C/B}$-H), 7.03 (d, 1H, ^3J = 6.4 Hz, 6'-H), 7.07 (d, 1H, ^3J = 6.6 Hz, 6'-H), 7.11 (d, 1H, ^3J = 7.2 Hz, 6'-H), 7.14 – 7.28 (m, 30H, Phe^{A}-H$_{ar}$, Phe^{B}-H$_{ar}$, Phe^{C}-H$_{ar}$), 7.29 – 7.36 (m, 3H, 4$^{A'}$-H, 4$^{B'}$-H, 4$^{C'}$-H), 7.41 – 7.47 (m, 3H, 5$^{A'}$-H, 5$^{B'}$-H, 5$^{C'}$-H), 7.62 – 7.68 (m, 3H, 3$^{A'}$-H, 3$^{B'}$-H, 3$^{C'}$-H), 8.03 – 8.18 (m, 7H, Phe^{3A}-NH, Phe^{3B}-NH, Phe^{3C}-NH, Phe^{4A}-NH, Phe^{4B}-NH, Phe^{4C}-NH, Ala-NH), 8.20 – 8.28 (m, 4H, 2x Ala –NH, 6^{B}-NH, 6^{C}-NH), 8.44 – 8.56 (m, 3H, Val^{A}-NH, Val^{B}-NH, Val^{C}-NH), 8.84 – 8.88 (m, 3H, N=CH^{A}, N=CH^{B}, N=CH^{C}).

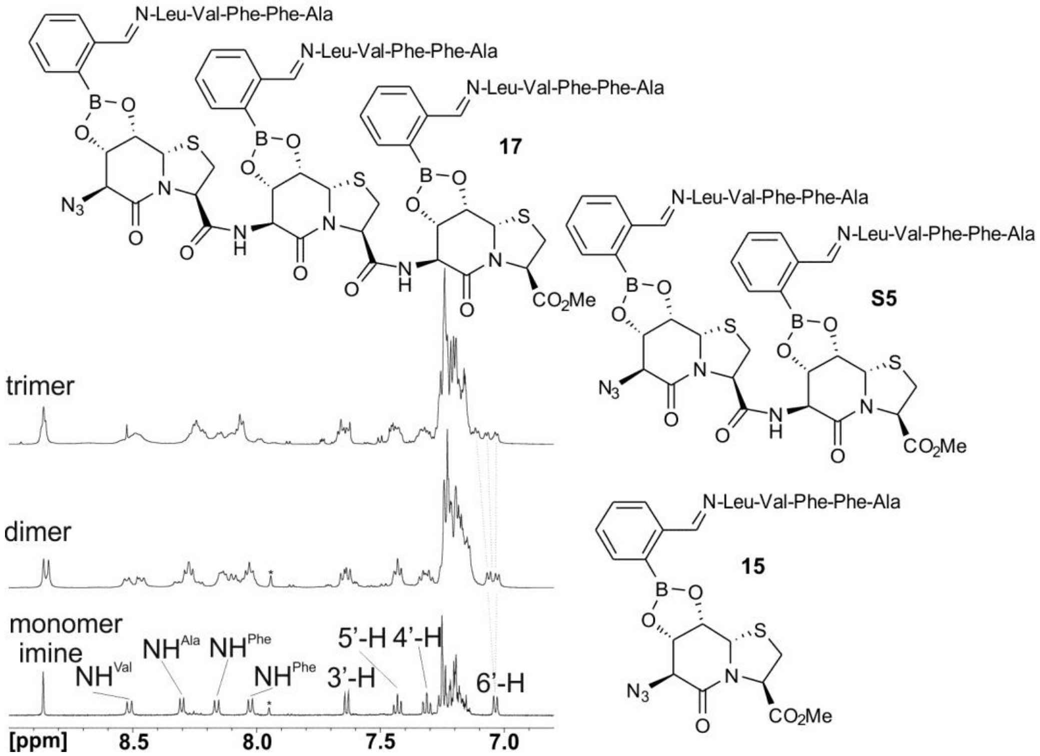

Figure S4: Expansions from the ^{1}H NMR spectra of monomeric (**5**), dimeric (**9**), and trimeric (**16**) template, with peptide Leu-Val-Phe-Phe-Ala and 2-formylphenylboronic acid (**14**), respectively, in DMSO-d6 (500, 600 MHz, 300 K) yielding esters **15**, **S5**, and **17**.
